# Rough-Fuzzy Clustering and Unsupervised Feature Selection for Wavelet Based MR Image Segmentation

**DOI:** 10.1371/journal.pone.0123677

**Published:** 2015-04-07

**Authors:** Pradipta Maji, Shaswati Roy

**Affiliations:** Biomedical Imaging and Bioinformatics Lab, Machine Intelligence Unit, Indian Statistical Institute, 203 B. T. Road, Kolkata, 700 108, India; University of Minnesota, UNITED STATES

## Abstract

Image segmentation is an indispensable process in the visualization of human tissues, particularly during clinical analysis of brain magnetic resonance (MR) images. For many human experts, manual segmentation is a difficult and time consuming task, which makes an automated brain MR image segmentation method desirable. In this regard, this paper presents a new segmentation method for brain MR images, integrating judiciously the merits of rough-fuzzy computing and multiresolution image analysis technique. The proposed method assumes that the major brain tissues, namely, gray matter, white matter, and cerebrospinal fluid from the MR images are considered to have different textural properties. The dyadic wavelet analysis is used to extract the scale-space feature vector for each pixel, while the rough-fuzzy clustering is used to address the uncertainty problem of brain MR image segmentation. An unsupervised feature selection method is introduced, based on maximum relevance-maximum significance criterion, to select relevant and significant textural features for segmentation problem, while the mathematical morphology based skull stripping preprocessing step is proposed to remove the non-cerebral tissues like skull. The performance of the proposed method, along with a comparison with related approaches, is demonstrated on a set of synthetic and real brain MR images using standard validity indices.

## Introduction

Magnetic resonance imaging (MRI) is an important diagnostic imaging technique for the early detection of abnormal changes in tissues and organs, and therefore, majority of research in medical imaging concerns MR images [1]. Conventionally, the brain MR images are interpreted visually and qualitatively by radiologists. Advanced research requires quantitative information such as the size of the brain tumor or brain ventricles after a traumatic brain injury or the relative volume of ventricles to brain. Fully automatic methods sometimes fail, producing incorrect results and requiring the intervention of a human operator. This is often true due to restrictions imposed by image acquisition, pathology and biological variation. So, it is important to have a faithful method to measure various structures in the brain.

Image segmentation is a process of partitioning an image space into some non-overlapping meaningful homogeneous regions. The success of an image analysis system depends on the quality of segmentation [1–3]. In the analysis of medical images for computer-aided diagnosis and therapy, segmentation is often required as a preliminary stage. The brain has a particularly complicated structure and its precise segmentation is very important for detecting tumors, edema, and necrotic tissues, in order to prescribe appropriate therapy [1–3]. Thresholding is one of the old, simple, and popular techniques for image segmentation. A series of algorithms for image segmentation based on histogram thresholding can be found in the literature [4–10]. However, the segmentation of brain MR image by thresholding the image intensities is difficult due to the fact that the T1 weighted MR image with contrast enhancement is the standard modality for identifying different regions in brain. The information provided by the intensities in this modality is not always consistent, and it is generally impossible to segment brain MR image by thresholding the intensities in this image modality.

The major aim of any image processing or analysis research is to develop better tools that may extract different perspectives on the same image, to understand not only its content, but also its meaning and significance. Texture is a fundamental characteristic of an image and plays an important role in the human visual system for recognition and interpretation of images. The analysis of image texture content is extremely important in image analysis. It requires the understanding of how humans discriminate between different texture types and how to model algorithms to perform image analysis task in a best possible way. Texture is an important property of all reflective natural surfaces which helps human visual perception system to segment and classify different objects in a digital image.

In a medical image, texture can be considered to be the visual impression of coarseness or smoothness caused by the variability or uniformity of image tone. These textural properties of a brain MR image are likely to provide valuable information for classification or segmentation, where different object regions are treated as different texture classes, that is, a multitexture segmentation problem. The segmentation of these images is necessary in order to identify different meaningful regions. Also, there is a change in appearance of most textures when viewed at different resolutions, and during the empirical division from macro to micro textures. Texture can also be defined as a local statistical distribution of pixel pattern (micro region) in observer’s domain [11–15]. Psychovisual studies reveal that the human visual system processes images in multiple scales. The visual cortex has separate cells that decomposes images into filtered images of various band of frequencies and orientation; thus capable of preserving both local and global information. Hence, the methods for texture analysis, based on the concept of multiscale processing of the human visual system, are superior over the more traditional ones. Texture is especially suited for this type of multiresolution analysis, using both frequency and spatial information due to its inherent characteristics.

Brain MR images may contain information over a large range of scales and the spatial frequency structure also changes over different regions, that is, non-periodic signal. In medical imaging perspective, the resolution of the imagery may be different in many cases, and so it is important to understand how information changes over different scales of imagery. The wavelet based multiresolution analysis is most effective for this purpose [16–18]. Moreover, wavelet theory is well suited in this area of study where signals are complex and non-periodic. Furthermore, wavelets are particularly good in describing a scene in terms of the scale of the textures in it [19, 20]. During the past two decades, wavelet analysis has become an important paradigm for multiresolution analysis, and has found important applications in image analysis [19–21]. However, the automatic brain MR image segmentation methods reported in [22–33] have not used multiresolution techniques to extract features. In [34], a method is reported to segment MR images, where discrete wavelet transform is used to extract high level details from MR image. The fuzzy *c*-means [35] is applied to the wavelet transformed image and Kirch’s edge detection mask is used to enhance the edge detail in the image. Finally, the resultant image is combined with the original image to get a sharpened image. Barra and Boire [36] proposed an algorithm for the segmentation of brain MR images, combining the merits of possibilistic *c*-means [37] and 3D wavelet analysis. While the fuzzy logic of possibilistic *c*-means algorithm models the uncertainty and imprecision inherent in brain MR images, the wavelet representation allows for both spatial and textural information.

The process of automatically extracting different regions of brain MR images is a challenging process due to the gradual transition between different classes of brain. This results in the ambiguity of the structural boundaries. Hence, one of the main problems in brain MR image segmentation is uncertainty. In this background, the rough-fuzzy computing provides a mathematical framework to capture uncertainties associated with human cognition process [38]. It is an efficient hybrid technique based on judicious integration of the principles of rough sets and fuzzy sets. Since the rough-fuzzy approach has the capability of providing a stronger paradigm for uncertainty handling, it has greater promise in application domains of pattern recognition and image processing, where fuzzy sets and/or rough sets are being effectively used and proved to be successful. The rough-fuzzy clustering algorithms such as rough-fuzzy *c*-means [39] and robust rough-fuzzy *c*-means [40] can avoid the problems of noise sensitivity of fuzzy *c*-means [35] and the coincident clusters of possibilistic *c*-means [37].

In this regard, the paper presents a texture-based brain MR image segmentation method, judiciously integrating the merits of multiresolution image analysis and rough-fuzzy computing. The proposed brain MR image segmentation is based on the assumption that different tissue classes of brain MR image belong to different texture categories. The multiresolution wavelet analysis is used to extract scale-space feature vector for each pixel of the given brain MR image. Since the boundary between brain and skull is relatively strong on T1 scan, a skull stripping algorithm is introduced to extract the brain tissues and remove non-cerebral tissues like skull. The skull stripped feature vectors are considered for more accurate segmentation. However, the use of wavelet decomposition may give rise to some irrelevant and insignificant features. Hence, the selection of appropriate features using some feature selection algorithms is required. In this background, an unsupervised feature selection method is proposed to reduce the dimensionality of feature space by maximizing both relevance and significance of the selected features. The measure of energy content of features is employed to compute both the relevance and significance. Finally, the rough-fuzzy clustering algorithm is used for segmentation of the given brain MR image. The rough-fuzzy clustering integrates judiciously the merits of rough sets, and probabilistic and possibilistic memberships of fuzzy sets. While the integration of both membership functions of fuzzy sets enables efficient handling of overlapping classes in noisy environment, the concept of lower and upper approximations of rough sets deals with uncertainty, vagueness, and incompleteness in class definition. In effect, it groups similar textured tissue classes contained in the image. The performance of the proposed approach, along with a comparison with related methods, is demonstrated on a set of synthetic and real brain MR images both qualitatively and quantitatively.

## Proposed Segmentation Methodology

**Fig 1:**
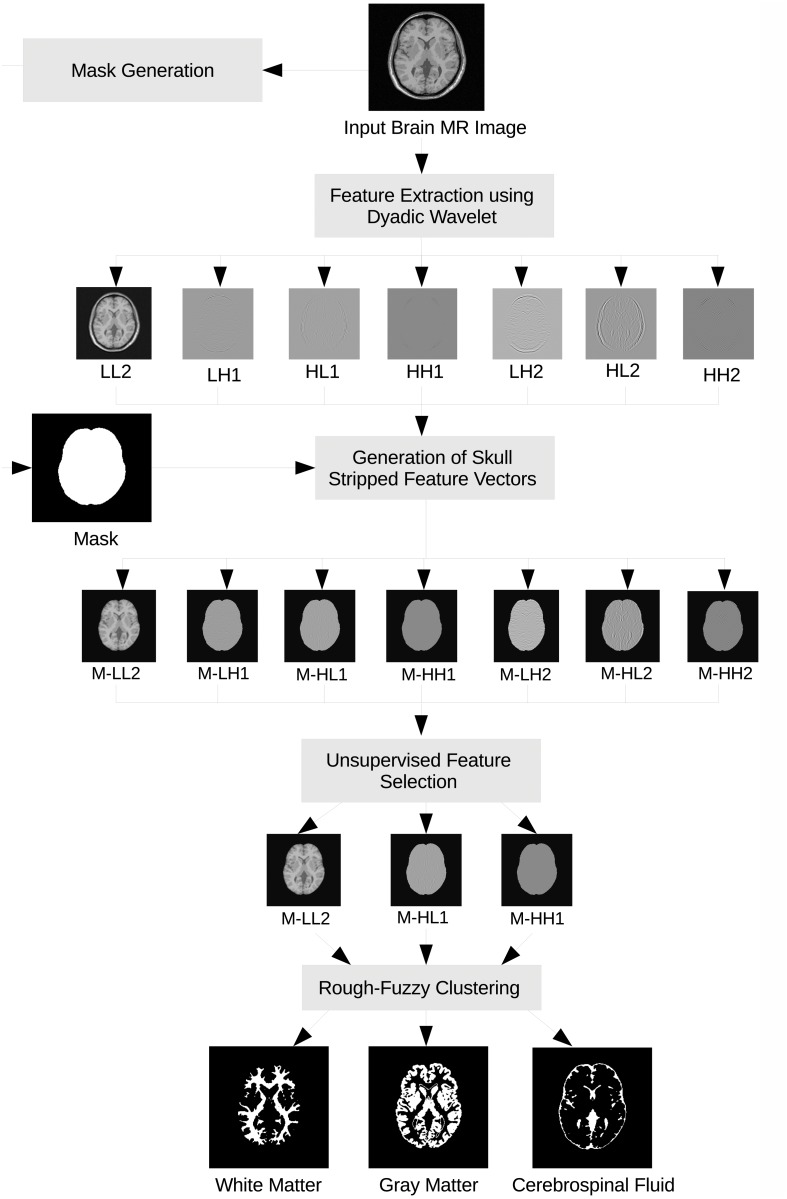
Block diagram of the proposed brain MR image segmentation method.

This section presents the proposed segmentation method in detail for brain MR image segmentation. It consists of mainly five steps as mentioned in Fig 1 and described below:

1. Generation of mask from the input MR image for identification of region of interest, that is, brain region;
2. Decomposition of the MR image using wavelet;
3. Generation of feature vectors for region of interest using the mask;
4. Unsupervised feature selection to select relevant and significant features for clustering; and
5. Rough-fuzzy clustering to generate segmented image.

Fig 1 presents the block diagram of the proposed segmentation method considering the brain MR image as an example. It also shows all the intermediate images obtained through the proposed technique. The subbands generated using wavelet decomposition are named as to reveal their orientation information. The approximation, horizontal, vertical, and diagonal subbands are denoted by LL, LH, HL, and HH, respectively, while these are followed by a number indicating the wavelet decomposition level.

Let the input brain MR image be *I* with size *M*×*N*. Hence, the total number of pixels is *n*_tot_ = *MN*. Let $\overset{´}{X}=\{x_{1},\cdots ,x_{i},\cdots ,x_{n_{\text{tot}}}\}$ be the set of pixels of the input image *I*. To identify region of interest, a mask is generated from input brain MR image using a new skull stripping algorithm. The algorithm is based on the thresholding method and mathematical morphology. After generating the mask, the input brain MR image is decomposed upto *l*th level using dyadic wavelets resulting into *d* = 3*l*+1 number of subbands. Hence, each pixel $x_{i}\in \overset{´}{X}$ of the input brain MR image is represented by *d* features. The mask is then applied to each of the subbands to generate the feature vectors for only brain region. In effect, a reduced set $X\subset \overset{´}{X}$ is generated considering only brain region, where *X* = {*x*_1_, ⋯, *x*_*i*_, ⋯, *x*_*n*_}, *n* < *n*_tot_ is the number of pixels within the region of interest. An unsupervised feature selection algorithm, based on maximum relevance-maximum significance criterion, is introduced to select *m* number of relevant and significant features from the whole set of *d* features for clustering. Finally, the rough-fuzzy clustering algorithm is used to generate the segmented image. Each step of the proposed segmentation method is elaborated next one by one.

### Identification of Region of Interest

The skull stripping is an important preprocessing phase in brain imaging application, which involves removal of non-cerebral tissues like skull, scalp, and dura from brain MR images. It reduces misclassification during segmentation as well as minimizes the execution time of segmentation algorithm by eliminating the objects for non-cerebral tissues [41].

Although many skull stripping techniques have been studied, most of them are based on either region growing techniques [42, 43] or mathematical morphology [44–46]. The success of skull removing methods using region growing approaches relies on the seed selection algorithm, which automatically selects seeds corresponding to the brain and non-brain regions. However, most of the brain MR image segmentation methods use morphological operations to separate the brain tissues from the surrounding tissues without holes, although morphology requires a prior binarization of the image and thresholding may be used to create binary images from gray level ones.

Since the morphology operations need binarization of the image, selection of a threshold value is crucial to generate the mask for brain tissues, in turns, to ensure the accurateness of the segmented results. The thresholding method used in this work is based on the mean image intensity value. On the other hand, the morphology operations use an octagonal shaped structuring element to produce the skull-stripped image. In the proposed brain MR image segmentation methodology, the skull stripping technique includes the following steps:

1. Apply median filtering with a window of size 3×3 to the input image.
2. Compute the initial mean intensity value *T*_*i*_ of the image.
3. Identify the top, bottom, left, and right pixel locations, from where brain skull starts in the image, considering gray values of the skull are greater than *T*_*i*_.
4. Form a rectangle using the top, bottom, left, and right pixel locations.
5. Compute the final mean value *T*_*f*_ of the brain using the pixels located within the rectangle.
6. Approximate the region of brain membrane or meninges that envelop the brain, based on the assumption that the intensity of skull is more than *T*_*f*_ and that of membrane is less than *T*_*f*_.
7. Set the average intensity value of membrane as the threshold value *T*.
8. Convert the given input image into binary image using the threshold *T*.
9. Apply a 13×13 opening morphological operation to the binary image in order to separate the skull from the brain completely.
10. Find the largest connected component and consider it as brain.
11. Finally, apply a 21×21 closing morphological operation to fill the gaps within and along the periphery of the intracranial region.

### Feature Extraction Using Multiresolution Wavelet Analysis

Wavelets mean small waves, that is, short duration finite energy functions. Wavelet function must be chosen from the space of all measurable functions that are absolutely and square integrable, that is, *L* ^1^(ℝ)∩*L* ^2^(ℝ). Being in this space a mother wavelet function *ψ*(*t*) must satisfy the conditions of zero mean and square norm, that is,

$$\begin{array}{c} \int_{-\infty }^{+\infty }\psi \left(t\right)dt=0<\infty \;\;\text{and}\int_{-\infty }^{+\infty }{\psi (t)}^{2}dt=1<\infty \cdot \end{array}$$ (1)

Mathematically, a wavelet is defined as

$$\begin{array}{c} \psi_{a,b}\left(t\right)=\frac{1}{\sqrt{a}}\psi (\frac{t-b}{a}) \end{array}$$ (2)

where *b* is the location parameter and *a* is the scaling parameter. The continuous wavelet transform is defined using mother wavelet *ψ*(*t*) as

$$\begin{array}{c} W\left(a,b\right)=\int_{t}f\left(t\right)\frac{1}{\sqrt{a}}\psi (\frac{t-b}{a})dt\cdot \end{array}$$ (3)

According to (3), for every (*a*, *b*), a wavelet transform coefficient is obtained, representing how much the scaled wavelet is similar to the function at location *t* = (*b*/*a*). The mother wavelet *ψ*(*t*) has to satisfy the admissibility condition

$$\begin{array}{c} C_{h}=\int_{-\infty }^{\infty }\frac{{\left|\Psi \left(w\right)\right|}^{2}}{\left|w\right|}dw<\infty \cdot \end{array}$$ (4)

In (4), Ψ(*w*) denotes Fourier transform of *ψ*(*t*). The admissibility condition assures that the function *f*(*t*) can be reconstructed with the knowledge of its wavelet transform by the following inverse transform relationship

$$\begin{array}{c} f\left(t\right)=\frac{1}{C_{h}}\int_{-\infty }^{\infty }\frac{1}{a^{2}}\int_{-\infty }^{\infty }W\left(a,b\right)\psi_{a,b}\left(t\right)dbda\cdot \end{array}$$ (5)

The multiresolution analysis is designed to provide good time resolution and poor frequency resolution at high frequencies and good frequency resolution and poor time resolution at low frequencies [16, 19, 20]. In multiresolution analysis, a scaling function is used to create a series of approximations of a function or image, each differing by a factor of 2 from its nearest neighboring approximation. Additional functions, called wavelets, are then used to encode the difference in information between adjacent approximations. Scaling function *ϕ*(*t*) and wavelet function *ψ*(*t*) are defined as follows:

$$\begin{array}{c} \Phi_{j,k}\left(t\right)=2^{j/2}\Phi \left(2^{j}t-k\right); \end{array}$$ (6)

$$\begin{array}{c} \psi_{j,k}\left(t\right)=2^{j/2}\psi \left(2^{j}t-k\right); \end{array}$$ (7)

for all *j*, *k* ∈ ℤ. Here *k* determines the position along *x*-axis; and *j* determines function’s width, that is, how broad or narrow it is along *x*-axis. The scaling function *ϕ*(*t*) and wavelet function *ψ*(*t*) are chosen appropriately to satisfy the orthonormality condition. The wavelet subspaces *W*_*j*_s form an orthogonal decomposition of *L* ^2^(ℝ) function space and hence they are related to nested subspaces *V*_*j*_s as follows:

$$\begin{array}{c} V_{j-1}=V_{j-2}+W_{j-2};\;\;\;\;\;\;\;\;\;\;\;\;\;\;\;\;\;\\ V_{j}=V_{j-1}+W_{j-1}=V_{j-2}+W_{j-2}+W_{j-1}; \end{array}$$

where *V*_*j*_ is the function space spanned by *ϕ*_*j*, *k*_(*t*) over *k* and *W*_*j*_ is the function space spanned by *ψ*_*j*, *k*_(*t*).

If a function *f*(*t*) ∈ *L* ^2^(ℝ), its wavelet transform is

$$\begin{array}{c} W_{\Phi }\left(j_{0},k\right)=\left\langle f\left(t\right)\Phi_{j_{0},k}\left(t\right)\right\rangle =\frac{1}{\sqrt{N}}\sum_{t}f\left(t\right)\Phi_{j_{0},k}\left(t\right); \end{array}$$ (8)

$$\begin{array}{c} W_{\psi }\left(j,k\right)=\left\langle f\left(t\right)\psi_{j,k}\left(t\right)\right\rangle =\frac{1}{\sqrt{N}}\sum_{t}f\left(t\right)\psi_{j,k}\left(t\right); \end{array}$$ (9)

where *j*_0_ is an arbitrary starting scale and *N* is the number of samples taken from the signal. The *W*_*ϕ*_(*j*_0_, *k*)’s are called approximation or scaling coefficients and *W*_*ψ*_(*j*, *k*)’s are referred to as detail or wavelet coefficients. In fast wavelet transform, at scale *j*, the approximation and detail coefficients, *W*_*ϕ*_(*j*, *k*) and *W*_*ψ*_(*j*, *k*), respectively, can be computed by convolving the scale *j*+1 approximation coefficients, *W*_*ϕ*_(*j*+1, *k*), with the time-reversed scaling and wavelet vectors, *h*_*ϕ*_(−*n*) and *h*_*ψ*_(−*n*), respectively, and subsampling the results.

Like one dimensional wavelet transform, the two dimensional wavelet transform can be implemented using the separable two dimensional scaling and wavelet functions. It is done by taking one dimensional wavelet transform of the rows of two dimensional function or image, followed by one dimensional wavelet transform of the resulting columns. Hence, it generates four subbands at each level, namely, approximation, horizontal, vertical, and diagonal subbands. The approximation part is iteratively decomposed as the decomposition level is increased in case of standard wavelet transform. Hence, if an input image is decomposed upto *l*th level, total *d* = 3*l*+1 number of subbands are generated.

The classical wavelet transform includes downsampling operations by a factor that cause wavelet expansions to be shift-variant. But, overcomplete representation of wavelets overcomes the shift-varying nature of classical wavelet expansion. Additionally, the overcomplete wavelet transform is convenient over the subsampled methods as downsampling decreases the size of the subbands at each increasing level of decomposition and thus may bias the decomposition at higher levels. Hence, the proposed methodology includes feature-extraction scheme that uses multiresolution dyadic wavelet filtering without downsampling.

### Generation of Skull Stripped Feature Vectors

After generating *d* subbands or features for a given image *I*, the mask is applied to each subband to generate the feature vectors for the region of interest only. Hence, a reduced set *X* = {*x*_1_, ⋯, *x*_*i*_, ⋯, *x*_*n*_} is generated from $\overset{´}{X}$ consisting of pixels within the region of interest. Each pixel or object *x*_*i*_ ∈ *X* is represented by *d*-dimensions, each dimension corresponding to each subband generated from wavelet decomposition. Next section presents an unsupervised feature selection algorithm, which is used to select *m* relevant and significant subbands or features from the whole set of *d* features for efficient segmentation.

### Unsupervised Feature Selection

In wavelet-based image segmentation method, a number of insignificant and irrelevant features may be generated. The presence of such features may lead to a reduction in the valuable information of segmentation. The objective of the feature selection is to identify a reduced feature subset with optimum salient characteristics of the image. It not only decreases the processing time, but also leads to more compactness and better generalization. The selected features should have high relevance and high significance in the feature set. In effect, these features will be able to predict the belongingness of the objects or samples in different segmented regions. However, as the segmentation problem is unsupervised in nature, the feature selection method should be unsupervised. Accordingly, a measure is required that can efficiently assess the effectiveness of feature set in unsupervised manner.

There exist many approaches to select a reduced set of relevant subbands for texture segmentation [47–51]. While Coifman and Wickerhauser [47] proposed entropy for the selection of best subbands, Saito et al. [48] used empirical probability density estimation and a local basis library for the extraction of discriminant features. On the other hand, Huang and Aviyente used mutual information in [50] for computing dependence among subbands to discard redundant or insignificant features, while both dependence and energy measures are used in [52] to select relevant and nonredundant subbands for texture classification. In the proposed feature selection algorithm, the energy measure is used to identify the relevant and significant features. The energy content of a feature set 𝕊 is defined as follows:

$$\begin{array}{c} ℰ\left(𝕊\right)=\frac{1}{n|𝕊|}\sum_{i=1}^{n}\sum_{j=1}^{\left|𝕊\right|}{\left(x_{ij}-\bar{x}\right)}^{2}=\frac{1}{n|𝕊|}\sum_{i=1}^{n}\sum_{j=1}^{\left|𝕊\right|}(x_{ij}^{2}-{\bar{x}}^{2}) \end{array}$$ (10)

as $\bar{x}$ is the mean of feature vectors of all objects and is given by

$$\begin{array}{c} \bar{x}=\frac{1}{n|𝕊|}\sum_{i=1}^{n}\sum_{j=1}^{\left|𝕊\right|}x_{ij}, \end{array}$$ (11)

where *n* is the number of objects and 𝕊 is the feature set. Hence, the energy content provides higher values for the features having high frequency components, indicating that these features contain more information. On the other hand, the energy content measure yields low energy values for the smoothed subbands indicating textural uniformity. Also, this measure is unsupervised in nature as it does not require any class information. Hence, this measure can be used to select potential wavelet features for image segmentation.

Let ℂ = {𝒜_1_, ⋯, 𝒜_*i*_, ⋯, 𝒜_*j*_, ⋯, 𝒜_*d*_} be the set of *d* features and 𝕊 is the set of selected features. The relevance *γ*_𝒜_*i*__ of the feature 𝒜_*i*_ is defined as

$$\begin{array}{c} \gamma_{{𝒜}_{i}}=ℰ\left(\left\{{𝒜}_{i}\right\}\right)\cdot \end{array}$$ (12)

The significance *σ*_𝒜_*j*__({𝒜_*i*_, 𝒜_*j*_}) of the feature 𝒜_*j*_ with respect to the feature set {𝒜_*i*_, 𝒜_*j*_} defines the extent to which the feature 𝒜_*j*_ is contributing in the energy estimation computed using (10). The change in energy estimation when a feature is removed from the feature set, is the measure of the significance of the feature, and is given as

$$\begin{array}{c} \sigma_{{𝒜}_{j}}\left(\left\{{𝒜}_{i},{𝒜}_{j}\right\}\right)=\gamma_{\left\{{𝒜}_{i},{𝒜}_{j}\right\}}-\gamma_{{𝒜}_{i}}=ℰ\left(\left\{{𝒜}_{i},{𝒜}_{j}\right\}\right)-ℰ\left(\left\{{𝒜}_{i}\right\}\right)\cdot \end{array}$$ (13)

The higher the change in energy estimation, the more significant the feature is. If the significance is 0, then the feature is dispensable.

Therefore, the problem of selecting a set 𝕊 of relevant and significant features from the whole set ℂ of *d* features is equivalent to maximizing both the total relevance of all selected features and the total significance among the selected features. To solve the above problem satisfying maximum relevance-maximum significance (MRMS) criterion [53], the following greedy algorithm can be used:

1. Initialize ℂ ← {𝒜_1_, ⋯, 𝒜_*i*_, ⋯, 𝒜_*d*_} and 𝕊 ← ∅.
2. Calculate the relevance *γ*_𝒜_*i*__ of each feature 𝒜_*i*_ ∈ ℂ.
3. Select the feature 𝒜_*i*_ as the most relevant feature that has the highest relevance value *γ*_𝒜_*i*__. In effect, 𝕊 ← 𝕊∪{𝒜_*i*_} and ℂ ← ℂ\{𝒜_*i*_}.
4. Repeat the following two steps until the desired number of features *m* is selected.
5. Calculate the significance of each of the remaining features of ℂ with respect to the already selected features of 𝕊 and remove it from ℂ if it has zero significance value with respect to any one of the selected features.
6. From the remaining features of ℂ, select feature 𝒜_*j*_ that maximizes the following condition:
  $$\begin{array}{c} \gamma_{{𝒜}_{j}}+\frac{1}{\left|𝕊\right|}\sum_{{𝒜}_{i}\in 𝕊}\sigma_{{𝒜}_{j}}\left(\left\{{𝒜}_{i},{𝒜}_{j}\right\}\right);\;\;\;\text{or}\;\;\;ℰ\left(\left\{{𝒜}_{j}\right\}\right)+\frac{1}{\left|𝕊\right|}\sum_{{𝒜}_{i}\in 𝕊}[ℰ\left(\left\{{𝒜}_{i},{𝒜}_{j}\right\}\right)-ℰ\left(\left\{{𝒜}_{i}\right\}\right)]\cdot \end{array}$$ (14)
  As a result of that, 𝕊 ← 𝕊∪{𝒜_*j*_} and ℂ ← ℂ\𝒜_*j*_.

The MRMS criterion based feature selection algorithm reported above is unsupervised in nature since it does not require any a priori knowledge about the segmented regions of brain MR image. The algorithm runs until the desired number of features *m* is selected. In practice, we find that the following definition works well:

$$\begin{array}{c} m=\lceil \sqrt{d}\rceil \end{array}$$ (15)

where *d* represents the total number of features or subbands corresponding to the given image.

### Rough-Fuzzy Clustering for Segmentation

In the proposed method, the robust rough-fuzzy *c*-means (rRFCM) [40] algorithm is used for segmentation of brain MR images. However, other clustering algorithms such as hard *c*-means [54], fuzzy *c*-means [35], and rough-fuzzy *c*-means (RFCM) [39] can also be used for this purpose. The rRFCM adds the concepts of fuzzy memberships, both probabilistic and possibilistic, of fuzzy sets, and lower and upper approximations of rough sets into *c*-means algorithm. While the integration of both probabilistic and possibilistic memberships of fuzzy sets enables efficient handling of overlapping clusters in noisy environment, the rough sets deal with uncertainty, vagueness, and incompleteness in cluster definition.

Let *X* = {*x*_1_, ⋯, *x*_*j*_, ⋯, *x*_*n*_} be the set of *n* objects and *V* = {*v*_1_, ⋯, *v*_*i*_, ⋯, *v*_*c*_} be the set of *c* centroids, where *x*_*j*_ ∈ ℜ^*m*^ and *v*_*i*_ ∈ ℜ^*m*^. In the rRFCM, each of the clusters *β*_*i*_ is represented by a cluster center *v*_*i*_, a possibilistic lower approximation $\underline{A}(\beta_{i})$ and a probabilistic boundary region $B(\beta_{i})=\{\bar{A}(\beta_{i})\backslash \underline{A}(\beta_{i})\}$, where $\bar{A}(\beta_{i})$ denotes the upper approximation of cluster *β*_*i*_. According to the definitions of lower approximation and boundary of rough sets [55], if an object $x_{j}\in \underline{A}(\beta_{i})$, then $x_{j}\notin \underline{A}(\beta_{k}),\forall k\neq i$, and *x*_*j*_ ∉ *B*(*β*_*i*_), ∀*i*. That is, the object *x*_*j*_ is contained in *β*_*i*_ definitely. Hence, the memberships of the objects in lower approximation of a cluster should be independent of other centroids and clusters. Also, the objects in lower approximation should have different influence on the corresponding centroid and cluster. From the standpoint of compatibility with the cluster prototype, the membership of an object in the lower approximation of a cluster should be determined solely by how far it is from the prototype of the cluster, and should not be coupled with its location with respect to other clusters. As the possibilistic membership *ν*_*ij*_ given by (20) depends only on the distance of object *x*_*j*_ from cluster *β*_*i*_, it allows optimal membership solutions to lie in the entire unit hypercube rather than restricting them to the hyperplane given by (21).

On the other hand, if *x*_*j*_ ∈ *B*(*β*_*i*_), then the object *x*_*j*_ possibly belongs to cluster *β*_*i*_ and potentially belongs to other clusters. Hence, the objects in boundary regions should have different influence on the centroids and clusters, and their memberships should depend on the positions of all cluster centroids. So, in the rRFCM, the membership values of objects in lower approximation are identical to (20) of possibilistic *c*-means [37], while those in boundary region are the same as (19) of fuzzy *c*-means [35]. Hence, the rRFCM algorithm partitions *X* into *c* clusters by minimizing following objective function:

$$\begin{array}{c} J=\{\begin{array}{cc} \omega {𝒜}_{1}+\left(1-\omega \right){ℬ}_{1} & \text{if}\;\underline{A}\left(\beta_{i}\right)\neq \emptyset \text{,}\;B(\beta_{i})\neq \emptyset \\ {𝒜}_{1} & \text{if}\;\underline{A}\left(\beta_{i}\right)\neq \emptyset \text{,}\;B(\beta_{i})=\emptyset \\ {ℬ}_{1} & \text{if}\;\underline{A}\left(\beta_{i}\right)=\emptyset \text{,}\;B(\beta_{i})\neq \emptyset \end{array} \end{array}$$ (16)

$$\begin{array}{c} \text{where}\;\;{𝒜}_{1}=\overset{c}{\underset{i=1}{\sum }}\underset{x_{j}\in \underline{A}\left(\beta_{i}\right)}{\sum }{\left(\nu_{ij}\right)}^{{\overset{´}{m^{\prime }}}_{2}}||x_{j}-v_{i}|{|}^{2}+\overset{c}{\underset{i=1}{\sum }}\eta_{i}\underset{x_{j}\in \underline{A}\left(\beta_{i}\right)}{\sum }{\left(1-\nu_{ij}\right)}^{\overset{´}{{m^{\prime }}_{2}}}; \end{array}$$ (17)

$$\begin{array}{c} and\;\;{ℬ}_{1}=\overset{c}{\underset{i=1}{\sum }}\underset{x_{j}\in B\left(\beta_{i}\right)}{\sum }{\left(\mu_{ij}\right)}^{{\overset{´}{m}}_{1}}{\left\|x_{j}-v_{i}\right\|}^{2}. \end{array}$$ (18)

The parameters *ω* and (1−*ω*) correspond to the relative importance of lower and boundary regions, while $\overset{´}{m_{1}}\in [1,\infty )$ and $\overset{´}{m_{2}}\in [1,\infty )$ are the probabilistic and possibilistic fuzzifiers, respectively. The probabilistic *μ*_*ij*_ and possibilistic *ν*_*ij*_ membership functions are given by

$$\begin{array}{c} \mu_{ij}={\left[\sum_{k=1}^{c}{\left(\frac{\left|\right|x_{j}-v_{i}{\left|\right|}^{2}}{\left|\right|x_{j}-v_{k}{\left|\right|}^{2}}\right)}^{\frac{1}{{\overset{´}{m}}_{1}-1}}\right]}^{-1}; \end{array}$$ (19)

$$\begin{array}{c} \text{and}\;\;\nu_{ij}={\left[1+{\left\{\frac{\left|\right|x_{j}-v_{i}{\left|\right|}^{2}}{\eta_{i}}\right\}}^{\frac{1}{\left(\overset{´}{m_{2}}-1\right)}}\right]}^{-1}; \end{array}$$ (20)

$$\begin{array}{c} \text{subject}\;\text{to}\;\;\sum_{i=1}^{c}\mu_{ij}=1,\forall j,\;\;0<\sum_{j=1}^{n}\mu_{ij}<n,\forall i, \end{array}$$ (21)

$$\begin{array}{c} 0<\sum_{j=1}^{n}\nu_{ij}\leq n,\forall i;\;\text{and}\;\max_{i}\nu_{ij}>0,\forall j; \end{array}$$ (22)

where the scale parameter *η*_*i*_ is given by

$$\begin{array}{c} \eta_{i}=K\cdot \frac{\sum_{j=1}^{n}{\left(\nu_{ij}\right)}^{\overset{´}{m_{2}}}\left|\right|x_{j}-v_{i}{\left|\right|}^{2}}{\sum_{j=1}^{n}{\left(\nu_{ij}\right)}^{\overset{´}{m_{2}}}} \end{array}$$ (23)

which represents the zone of influence or size of the cluster *β*_*i*_. Typically, K is chosen to be 1. The centroid is calculated based on the weighting average of the possibilistic lower approximation and probabilistic boundary. The centroid calculation for the rRFCM is as follows:

$$\begin{array}{c} v_{i}=\{\begin{array}{cc} \omega {𝒞}_{1}+\left(1-\omega \right){𝒟}_{1} & \text{if}\;\underline{A}\left(\beta_{i}\right)\neq \emptyset \text{,}\;B(\beta_{i})\neq \emptyset \\ {𝒞}_{1} & \text{if}\;\underline{A}\left(\beta_{i}\right)\neq \emptyset \text{,}\;B(\beta_{i})=\emptyset \\ {𝒟}_{1} & \text{if}\;\underline{A}\left(\beta_{i}\right)=\emptyset \text{,}\;B(\beta_{i})\neq \emptyset \end{array} \end{array}$$ (24)

$$\begin{array}{c} \text{where}\;\;{𝒞}_{1}=\frac{\underset{x_{j}\in \underline{A}\left(\beta_{i}\right)}{\sum }{\left(\nu_{ij}\right)}^{{\overset{´}{m}}_{2}}x_{j}}{\underset{x_{j}\in \underline{A}\left(\beta_{i}\right)}{\sum }{\left(\nu_{ij}\right)}^{{\overset{´}{m}}_{2}}};\;\text{and}\;\;{𝒟}_{1}=\frac{\underset{x_{j}\in B\left(\beta_{i}\right)}{\sum }{\left(\mu_{ij}\right)}^{{\overset{´}{m}}_{1}}x_{j}}{\underset{x_{j}\in B\left(\beta_{i}\right)}{\sum }{\left(\mu_{ij}\right)}^{{\overset{´}{m}}_{1}}}\cdot \end{array}$$

The rRFCM algorithm starts by randomly choosing *c* objects as the centroids of the *c* clusters. The possibilistic memberships *ν*_*ij*_ of all the objects are calculated using (20). The scale parameters *η*_*i*_ for *c* clusters are obtained using (23). Let *ν*_*i*_ = (*ν*_*i*1_, ⋯, *ν*_*ij*_, ⋯, *ν*_*in*_) be the fuzzy cluster *β*_*i*_ associated with the centroid *v*_*i*_. After computing *ν*_*ij*_ for *c* clusters and *n* objects, the values of *ν*_*ij*_ for each object *x*_*j*_ are sorted and the difference of two highest memberships of *x*_*j*_ is compared with a threshold value *δ*_1_. Let *ν*_*ij*_ and *ν*_*kj*_ be the highest and second highest memberships of *x*_*j*_. If (*ν*_*ij*_−*ν*_*kj*_) > *δ*_1_, then $x_{j}\in \underline{A}(\beta_{i})$. In addition, by properties of rough sets, $x_{j}\in \bar{A}(\beta_{i})$. Otherwise, *x*_*j*_ ∈ *B*(*β*_*i*_) and *x*_*j*_ ∈ *B*(*β*_*k*_) if *ν*_*ij*_ > *δ*_2_. Furthermore, *x*_*j*_ is not part of any lower bound. After assigning each object in lower approximations or boundary regions of different clusters based on both *δ*_1_ and *δ*_2_, the memberships *μ*_*ij*_ for the objects lying in boundary regions are computed for *c* clusters using (19). The new centroids of different clusters are calculated as per (24). The above procedure is repeated until no more new assignments can be made.

The performance of the rRFCM depends on the values of two thresholds *δ*_1_ and *δ*_2_, which determine the cluster labels of all the objects. In other word, the rRFCM partitions the data set into two classes, namely, lower approximation and boundary, based on the values of *δ*_1_ and *δ*_2_. The thresholds *δ*_1_ and *δ*_2_ control the size of granules of rough-fuzzy clustering. In practice, the following definitions work well:

$$\begin{array}{c} \delta_{1}=\frac{1}{n}\sum_{j=1}^{n}\left(\nu_{ij}-\nu_{kj}\right) \end{array}$$ (25)

where *n* is the total number of objects, *ν*_*ij*_ and *ν*_*kj*_ are the highest and second highest memberships of object *x*_*j*_. That is, the value of *δ*_1_ represents the average difference of two highest possibilistic memberships of all the objects in the data set. A good clustering procedure should make the value of *δ*_1_ as high as possible. On the other hand, the objects with (*ν*_*ij*_−*ν*_*kj*_) ≤ *δ*_1_ are used to calculate the threshold *δ*_2_:

$$\begin{array}{c} \delta_{2}=\frac{1}{\overset{´}{n}}\sum_{j=1}^{\overset{´}{n}}\nu_{ij} \end{array}$$ (26)

where $\overset{´}{n}$ is the number of objects that do not belong to lower approximations of any cluster and *ν*_*ij*_ is the highest membership of object *x*_*j*_. That is, the value of *δ*_2_ represents the average of highest memberships of $\overset{´}{n}$ objects in the data set.

## Quantitative Indices

To evaluate the performance of different methods for segmentation of brain MR images, three quantitative measures, namely, Jaccard index, sensitivity, and specificity, are used. Let *A* and *B* be two sets representing the region of interest to be segmented in the ground truth or reference image and segmented image, respectively. Based on the ground truth image, the false positive (FP), false negative (FN), true positive (TP), and true negative (TN) counts can be computed for each segmented image.

The Jaccard similarity index measures the overlap between two sets *A* and *B*. It is defined as the size of the intersection of two sets divided by the size of their union, that is,

$$\begin{array}{c} J\left(A,B\right)=\frac{A\cap B}{A\cup B}\cdot \end{array}$$ (27)

Hence, it can also be expressed as

$$\begin{array}{c} J=\frac{\text{TP}}{\text{FP}+\text{TP}+\text{FN}}\cdot \end{array}$$ (28)

The Jaccard index is zero if the segment of interest in output image and the segment of corresponding features in ground truth image are disjoint, that is, they have no common data points, and is one if they are identical. Higher numbers represent better overlapping in these two segments, indicating the significance of underlying algorithm.

The sensitivity measures the fraction of true positives that are included in a segmentation, and is as follows:

$$\begin{array}{c} SN=\frac{\text{TP}}{\text{TP}+\text{FN}}\cdot \end{array}$$ (29)

A score of one of sensitivity indicates that all the points in the ground truth region of interest are included in the segmentation result. Since sensitivity does not include false positives or true negatives in its calculation, it does not indicate whether the region of interest in segmented result includes more than the corresponding ground truth region. Thus, sensitivity should generally not be used by itself to measure segmentation quality, specificity measure would be incorporated with it.

The specificity measures the fraction of pixels, that do not belong to the region of interest, correctly detected, as determined by the equation

$$\begin{array}{c} SP=\frac{\text{TN}}{\text{TN}+\text{FP}}\cdot \end{array}$$ (30)

## Experimental Results and Discussions

This section presents the performance of the proposed brain MR image segmentation method, along with a comparison with related methods. The proposed method integrates the merits of robust rough-fuzzy *c*-means (rRFCM) [40], dyadic wavelets, and proposed skull stripping and unsupervised feature selection algorithms. The source code of the proposed segmentation method, written in C language, is available at www.isical.ac.in/~bibl/results/bms/bms.html. The methods compared are ℳ_1_, ℳ_2_, ℳ_3_, ℳ_4_, ℳ_5_, ℳ_6_, ℳ_7_, ℳ_8_, FSL [56], and SPM [57] as described below:

1. ℳ_1_: Using proposed mask generation algorithm, gray value as feature, not using any feature selection algorithm, clustering using rRFCM;
2. ℳ_2_: Not using any mask, wavelet analysis for feature extraction, using proposed feature selection algorithm, clustering using rRFCM;
3. ℳ_3_: Using mask generated by brain extraction tool (BET) [41], wavelet analysis for feature extraction, using proposed feature selection algorithm, clustering using rRFCM;
4. ℳ_4_: Using proposed mask generation algorithm, wavelet analysis for feature extraction, not using any feature selection algorithm, clustering using rRFCM;
5. ℳ_5_: Using proposed mask generation algorithm, wavelet analysis for feature extraction, using feature selection algorithm proposed by Huang and Aviyente [50], clustering using rRFCM;
6. ℳ_6_: Using proposed mask generation algorithm, wavelet analysis for feature extraction, using proposed feature selection algorithm, clustering using hard *c*-means [54];
7. ℳ_7_: Using proposed mask generation algorithm, wavelet analysis for feature extraction, using proposed feature selection algorithm, clustering using fuzzy *c*-means [35];
8. ℳ_8_: Using proposed mask generation algorithm, wavelet analysis for feature extraction, using proposed feature selection algorithm, clustering using rough-fuzzy *c*-means [39];
9. FSL: a comprehensive library of analysis tools for MRI brain imaging data [56]; and
10. SPM: statistical parameter mapping (SPM) software version 8 [57].

**Fig 2:**
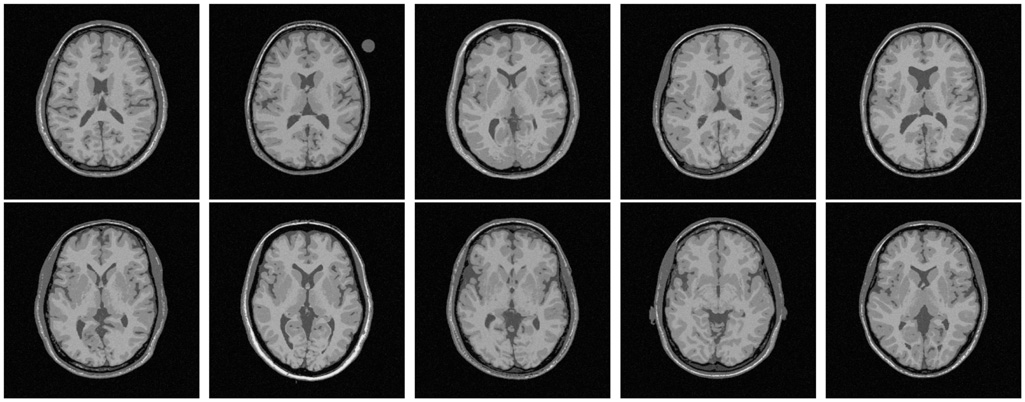
Original images of BrainWeb for subject no. 4, 5, 6, 43, 45, 47, 48, 49, 50, and 51.

**Fig 3:**
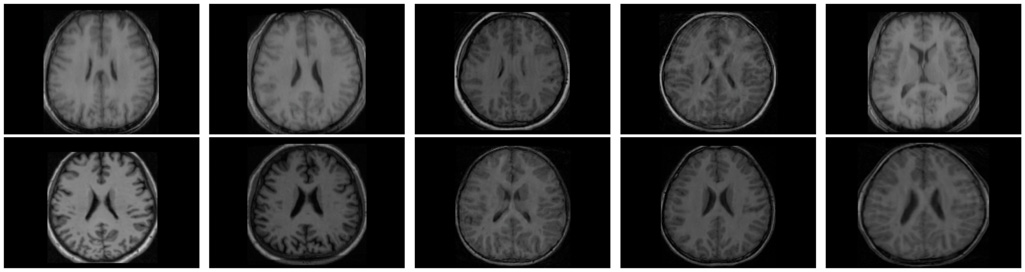
Original images of IBSR for volume no. 1, 2, 3, 4, 5, 11, 12, 13, 14, and 17.

**Fig 4:**
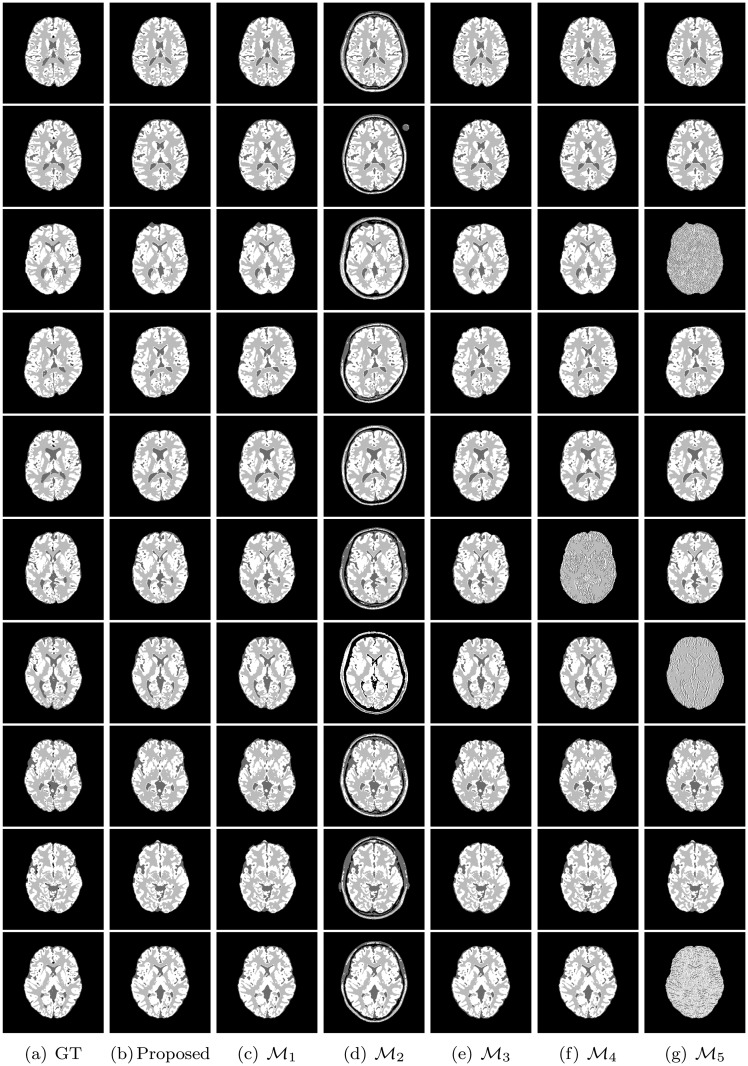
Ground truth (GT) and segmented images obtained using different methods on subject no. 4, 5, 6, 43, 45, 47, 48, 49, 50, and 51 of BrainWeb.

**Fig 5:**
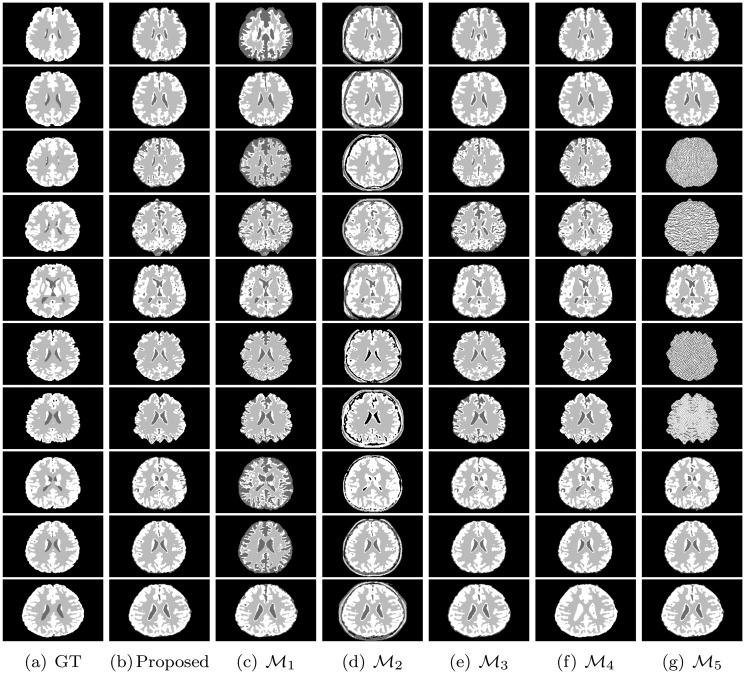
Ground truth (GT) and segmented images obtained using different methods on volume no. 1, 2, 3, 4, 5, 11, 12, 13, 14, and 17 of IBSR.

**Fig 6:**
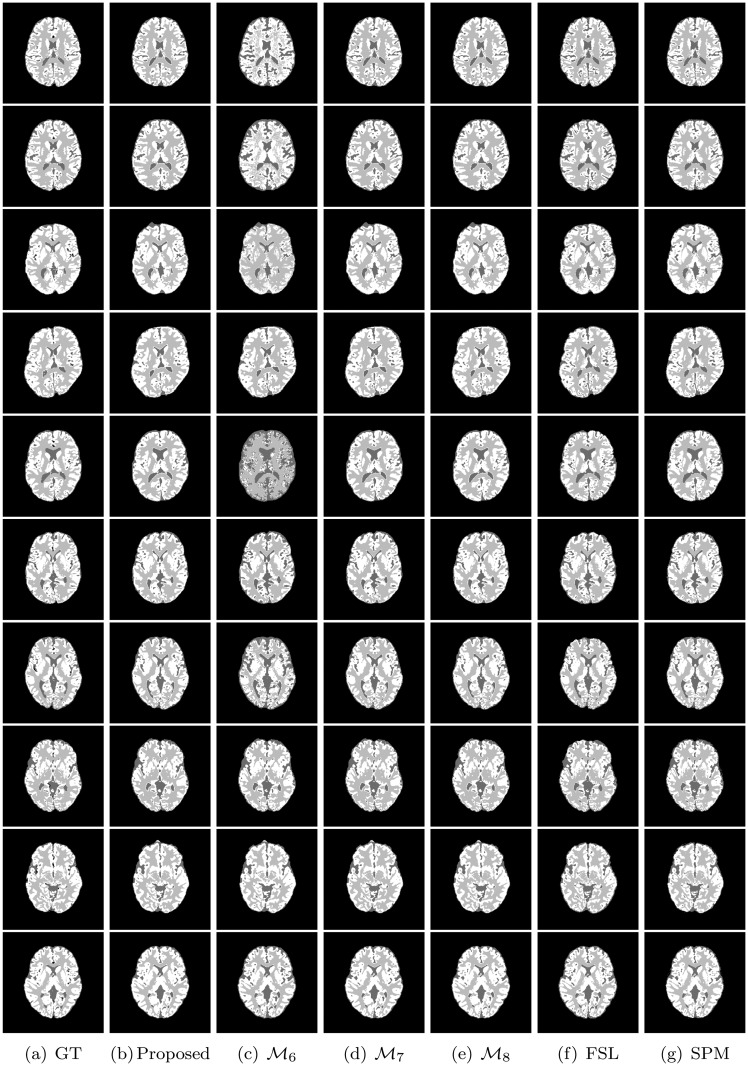
Ground truth (GT) and segmented images obtained using different methods on subject no. 4, 5, 6, 43, 45, 47, 48, 49, 50, and 52 of BrainWeb.

**Fig 7:**
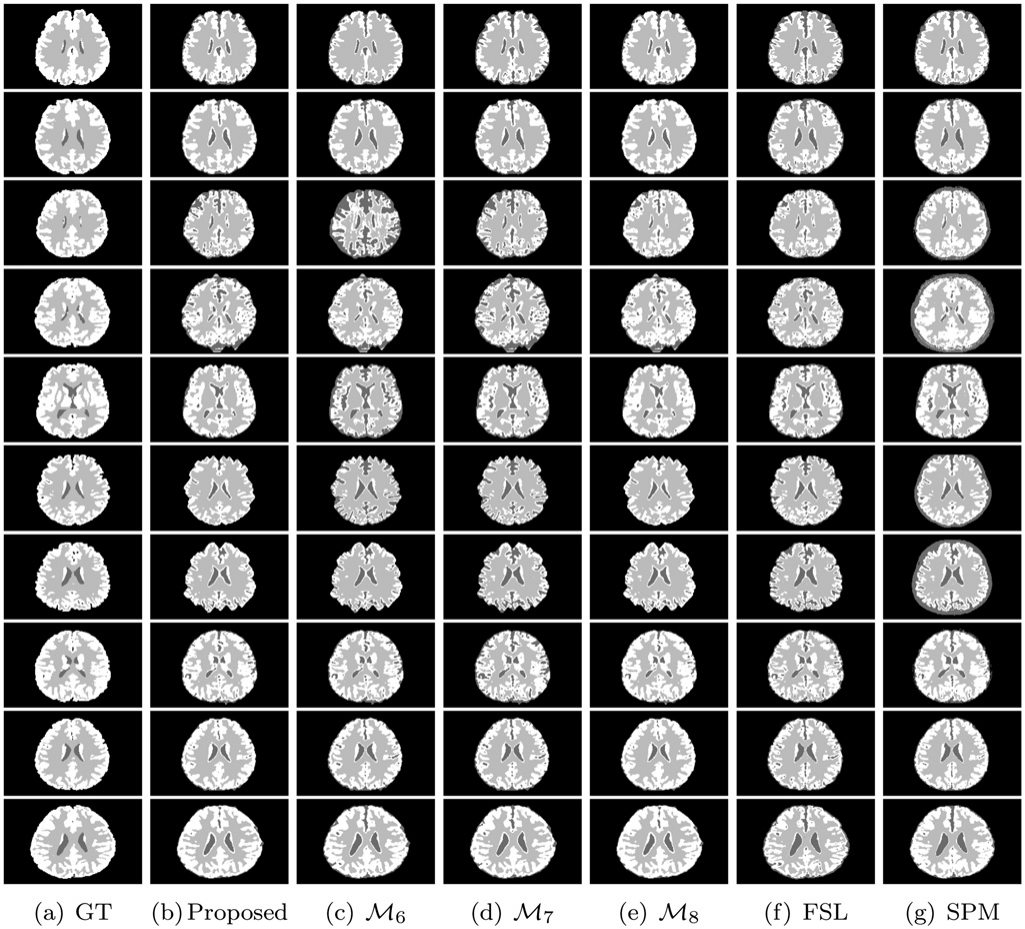
Ground truth (GT) and segmented images obtained using different methods on volume no. 1, 2, 3, 4, 5, 11, 12, 13, 14, and 17 of IBSR.

All the methods are implemented in C language and run in LINUX environment having machine configuration Intel(R) Core(TM) i7-2600 CPU @3.40GHz×8 and 16 GB RAM. To analyze the performance of different algorithms and measures, the experimentation is done on some benchmark simulated MR images obtained from “BrainWeb: Simulated Brain Database” (www.bic.mni.mcgill.ca/brainweb/) and real MR images of “IBSR: Internet Brain Segmentation Repository” (www.cma.mgh.harvard.edu/ibsr/). All the image volumes of BrainWeb and IBSR are of size 256×256×181 and 256×128×256, respectively. The middle slice of each volume is considered for both qualitative and quantitative analysis. Figs 2 and 3 depict some of the original images of BrainWeb and IBSR data sets, respectively, while Figs 4, 5, 6, and 7 present the segmented images obtained using different methods, along with the ground truth images. The first and second columns of Figs 4, 5, 6, and 7 show the ground truth images and output images obtained using the proposed method, while remaining columns present the segmented images produced by different methods.

In this regard, it should be noted that all the experiments are performed with no a priori knowledge about the input image. The comparative performance analysis is studied with respect to various segmentation metrics, namely, Jaccard index, sensitivity, and specificity. The metrics are calculated for individual tissue class and then averaged over all classes, that is, the identification of all tissue classes is given equal importance towards calculation of segmentation accuracy. Daubechies 6-tap filter is used to extract features using wavelet decomposition, where the filter coefficients are as follows:

**Table:** 

Number of Taps (*t*)	Scaling Function *ϕ*(*t*)	Wavelet Function *ψ*(*t*)
0	0.3326705530	-0.0352262919
1	0.8068915093	-0.0854412739
2	0.4598775021	0.1350110200
3	-0.1350110200	0.4598775021
4	-0.0854412739	-0.8068915093
5	0.0352262919	0.3326705530

The values of fuzzifiers $\overset{´}{m_{1}}=\overset{´}{m_{2}}=2\cdot 00$, while that of weight parameter *ω* for rough-fuzzy clustering algorithms is set to 0.99. The final cluster prototypes of hard *c*-means are used as the initial centroids of other clustering algorithms.

### Optimum Value of Wavelet Decomposition Level

The wavelet transform, at each level, generates four subbands, namely, approximation, horizontal, vertical, and diagonal. The approximation part is iteratively decomposed as the decomposition level is increased. Hence, if an input image is decomposed upto *l*th level, total (3*l*+1) number of subbands are generated. To find out the optimum value of decomposition level *l*, extensive experiments are carried out by varying *l* = 1 to 4 on several brain MR images.

**Fig 8:**
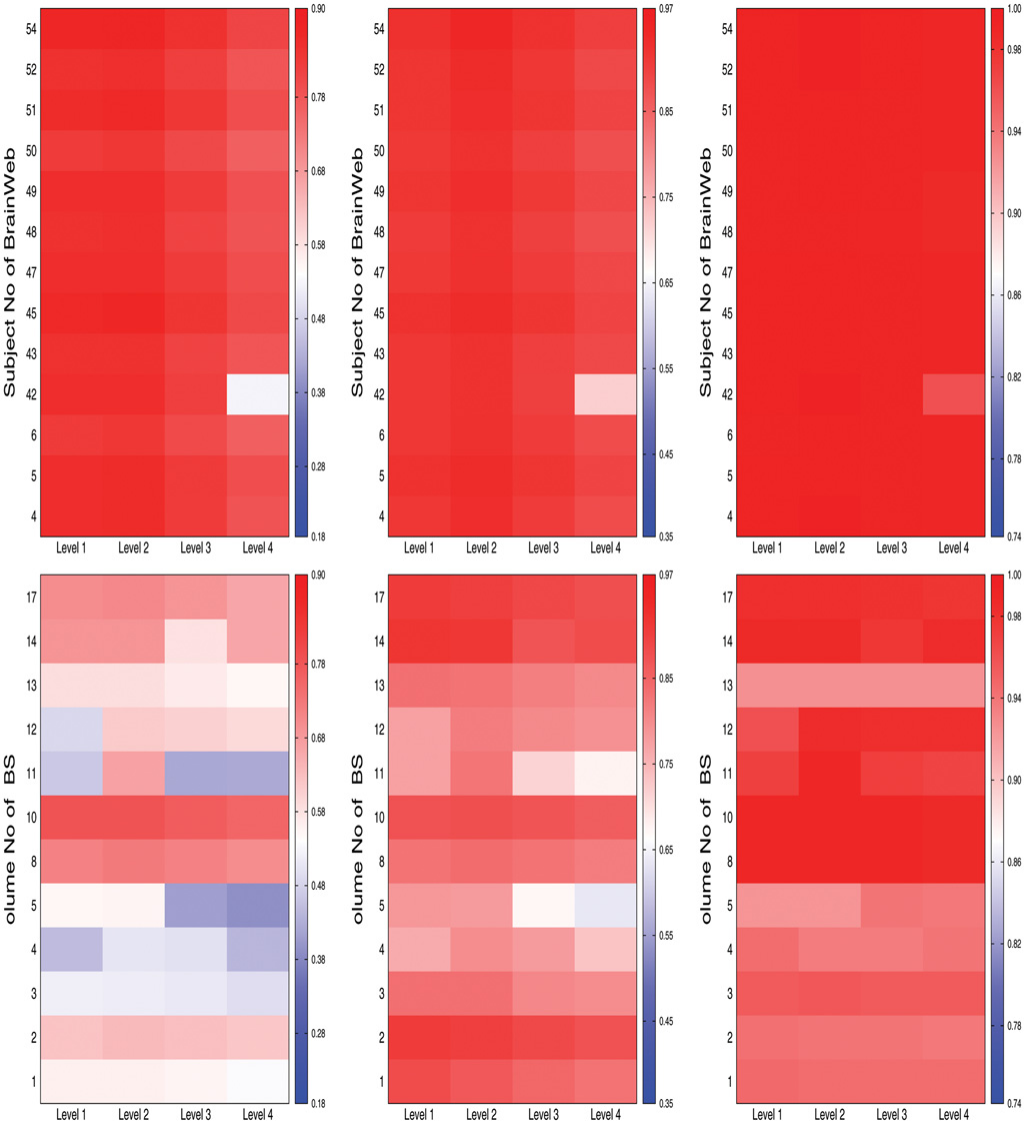
Heat maps for comparative performance analysis of different decomposition levels of wavelet analysis (from left to right: Jaccard index, sensitivity, and specificity).

Fig 8 reports the heat maps for comparative performance analysis of different decomposition levels with respect to Jaccard index, sensitivity, and specificity on both BrainWeb and IBSR data sets. From the results reported in Fig 8, it can be seen that the segmentation quality increases with the increase of decomposition level upto 2 irrespective of the segmentation metrics and data sets used. However, the performance of the proposed method detoriates for *l* ≥ 3. The proposed brain MR image segmentation method for *l* = 2 performs better than that of other levels in 24, 19, and 20 cases, out of 25 cases each, with respect to Jaccard index, sensitivity, and specificity, respectively. Out of total 75 cases, the proposed segmentation method with *l* = 2 provides better results in 63 cases, while that with *l* = 1 and *l* = 3 attains in only 10 and 2 cases, respectively. Hence, each image is decomposed upto second level without degrading the segmentation quality.

### Importance of Wavelet Based Feature Extraction Method

The proposed brain MR image segmentation method uses wavelet for feature extraction. The features are extracted using Daubechies 6-tap wavelet decomposing upto second level, which yields seven subbands as features. However, the proposed unsupervised feature selection algorithm reduces this dimension to three. To establish the importance of wavelet based analysis over gray level, that is, the performance of the proposed method over the method ℳ_1_, extensive experimentation is done on several brain MR images. The rRFCM algorithm is used as the clustering algorithm for both methods.

**Fig 9:**
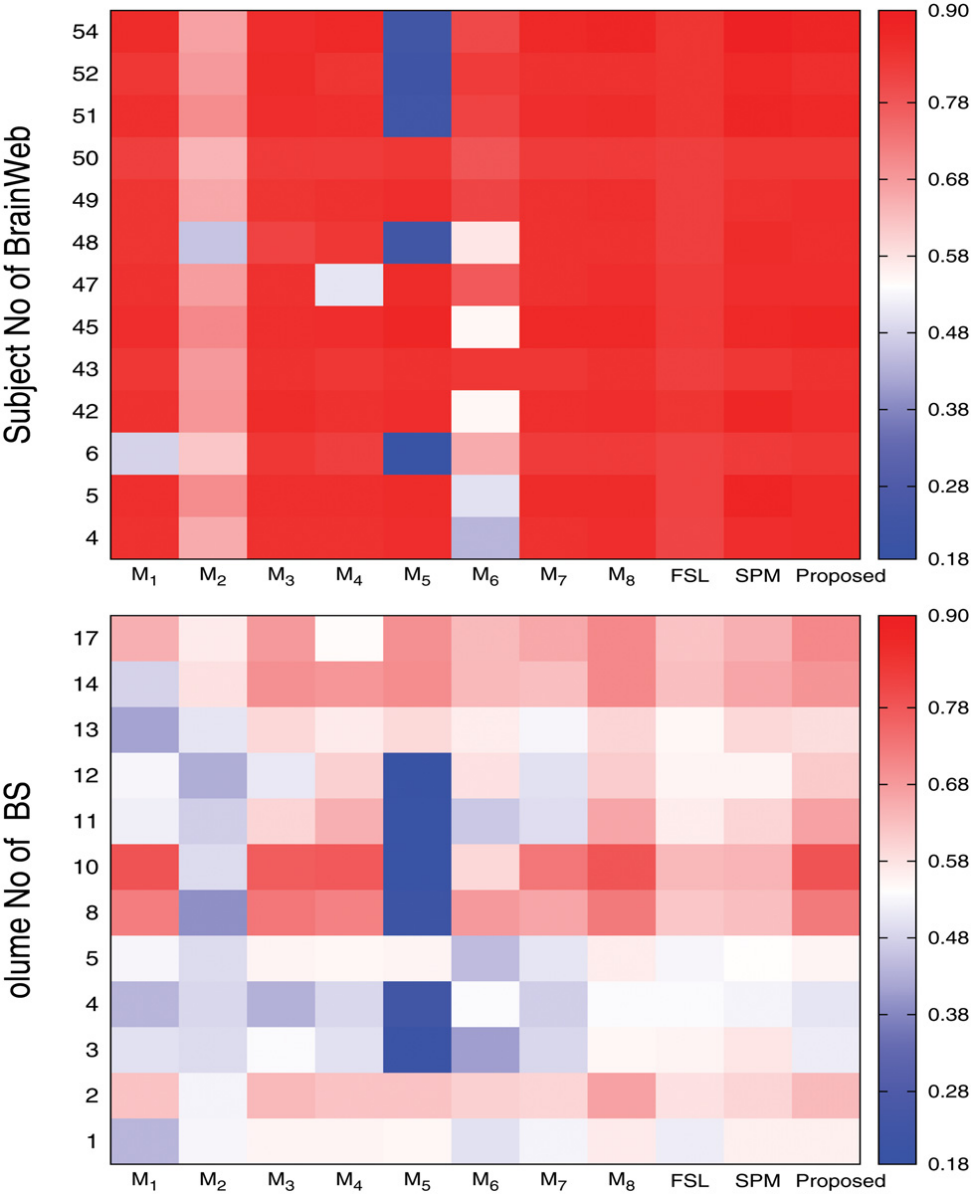
Heat maps obtained by different methods with respect to Jaccard index.

**Fig 10:**
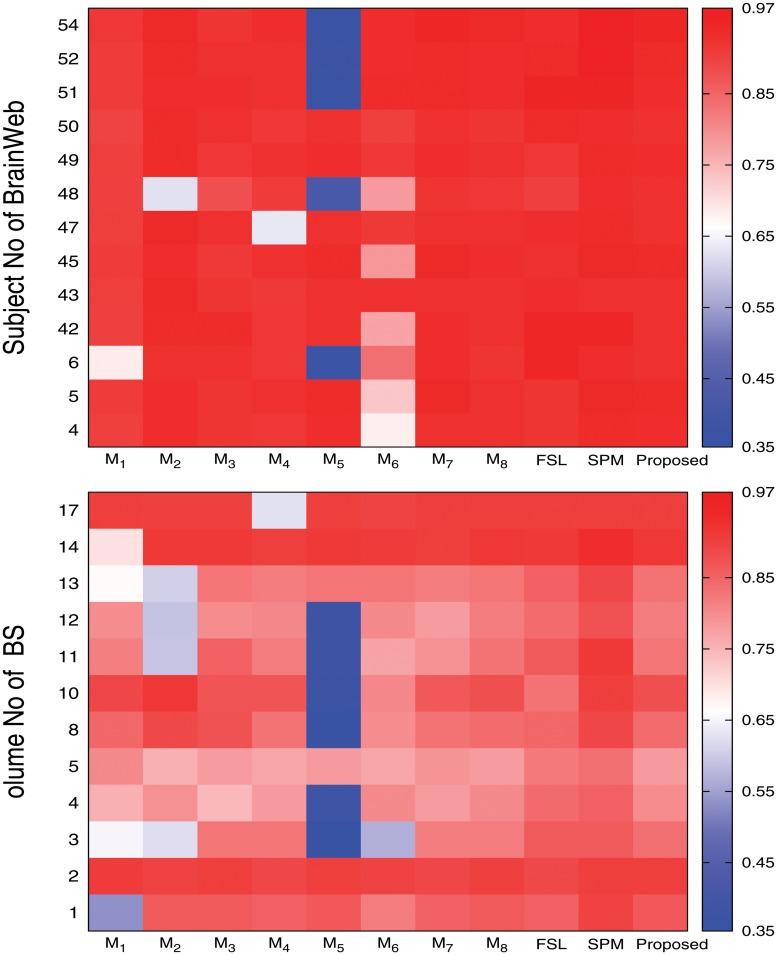
Heat maps obtained by different methods with respect to sensitivity.

**Fig 11:**
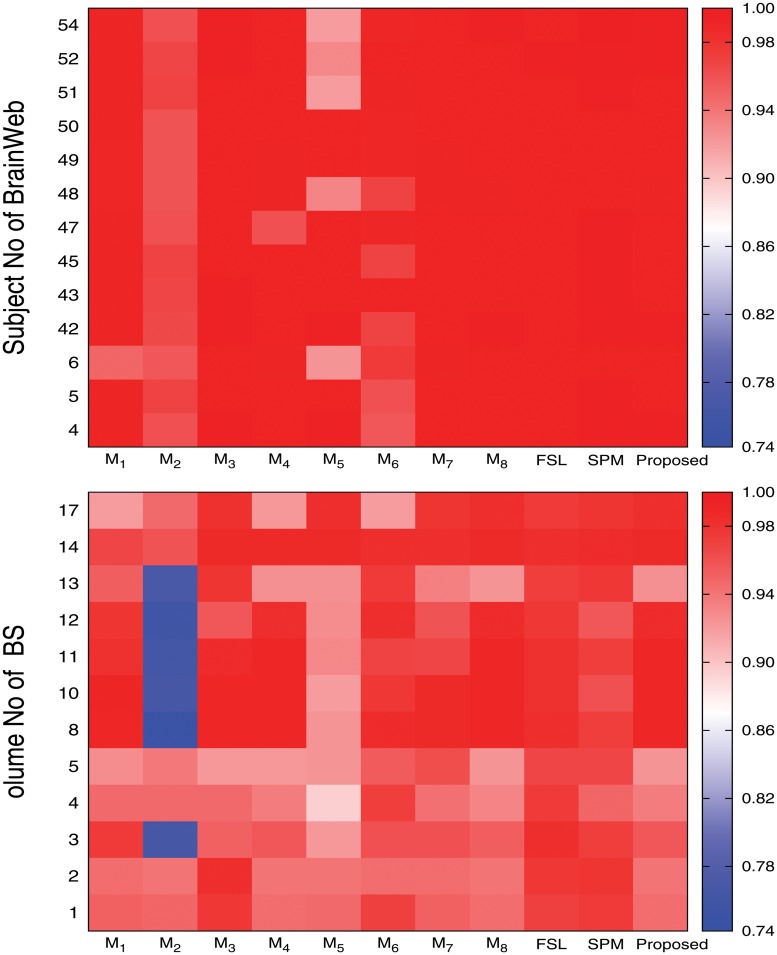
Heat maps obtained by different methods with respect to specificity.

Figs 9, 10, and 11 present the heat maps for comparative performance analysis of wavelet based feature extraction and gray level with respect to three quantitative indices, namely, Jaccard index, sensitivity, and specificity. From the results reported in Figs 9, 10, and 11, it can be seen that the performance of the proposed method is better than that of the ℳ_1_ in most of the cases, irrespective of the input images and quantitative indices used. Out of total 75 cases, the ℳ_1_ performs better than the proposed method in only 13 cases. The second and third columns of Figs 4 and 5 compare qualitatively the performance of wavelet based analysis and gray level, that is, the proposed and ℳ_1_ methods. All the results reported in second and third columns of Figs 4 and 5, and first and eleventh columns of heat maps presented in Figs 9, 10, and 11 confirm that the features derived by wavelet transform produce segmented images more promising than do the conventional gray level segmentation. The wavelet analysis provides a multiscale representation that resembles a hierarchical framework for interpreting the image information. At different scales, the details of an image generally characterize different physical structures of the image. In image processing, wavelet decomposition provides information from each scale to be analyzed separately. Hence, the feature extraction scheme based on multiscale analysis has several potential advantages over traditional gray level segmentation.

### Effectiveness of Skull Stripping Algorithm

**Fig 12:**
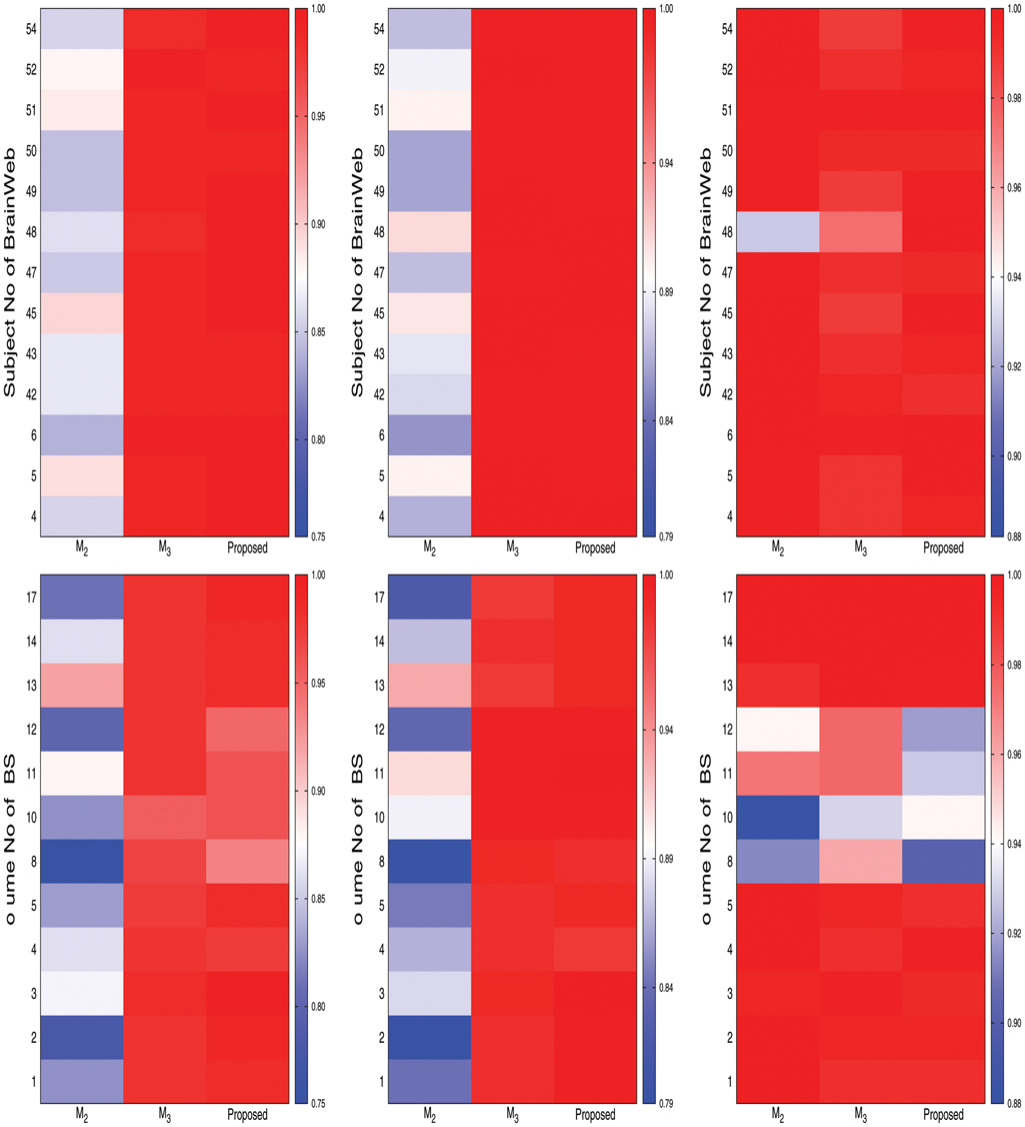
Heat maps for comparative performance analysis of the proposed method (skull stripping), the method ℳ_2_ (without skull stripping), and the method ℳ_3_ (masking using BET) for background separation (from left to right: Jaccard index, sensitivity, and specificity).

In the proposed segmentation method, the skull stripping algorithm is used to separate the background from major tissues of brain. To establish the effectiveness of the proposed skull stripping algorithm, the performance of the proposed method is compared with that of the methods ℳ_2_ and ℳ_3_. While second, third, and eleventh columns of heat maps presented in Figs 9, 10, and 11 compare the performance of the ℳ_2_, ℳ_3_, and the proposed method for three major tissue classes, namely, cerebrospinal fluid (CSF), gray matter, and white matter, Fig 12 presents that of only background region. From the results reported in Figs 9, 10, and 11, it can be seen that the proposed method with proposed skull stripping algorithm performs significantly better than the methods ℳ_2_ and ℳ_3_ for segmenting the CSF, gray matter, and white matter. The performance of the proposed method is better than that of ℳ_2_ in 25, 16, and 22 cases with respect to Jaccard index, sensitivity, and specificity, respectively, while the proposed skull stripping algorithm attains higher performance compared to the BET [41] of ℳ_3_ in 16, 21, and 13 cases, respectively.

The heat maps of Fig 12 compare the performance of the proposed method, ℳ_2_, and ℳ_3_ for segmentation of background region. From the results reported in Fig 12, it can be seen that the proposed method always attains better results than that of the ℳ_2_ with respect to Jaccard index and sensitivity. However, the specificity value of the proposed method is lesser as compared to that of the ℳ_2_ in most of the cases. Since the proposed skull stripping method adds some boundary portion of the CSF from brain image into its background, the false positive count is increased for the background. So, out of total 25 cases, the proposed method attains higher specificity values in only five cases compared to that of the ℳ_2_. Hence, the proposed method performs better than the ℳ_2_ in 55 cases out of total 75 cases, irrespective of the quantitative indices used. On the other hand, the proposed method provides better results than that of the ℳ_3_ in most of the cases irrespective of the quantitative indices and data sets used. In other words, the performance of the proposed skull stripping algorithm is better than that of the BET [41] in 18, 15, and 17 cases with respect to Jaccard index, sensitivity, and specificity, respectively. Considering all the results reported in Figs 9, 10, 11, and 12, out of total 150 cases, the proposed method attains best performance in 79 cases, irrespective of the quantitative indices and images used, while both ℳ_2_ and ℳ_3_ achieve it in only 26 and 45 cases, respectively. In Figs 4 and 5, the qualitative performance analysis of the proposed method, ℳ_2_, and ℳ_3_ is reported. All the results reported in second, fourth, and fifth columns of Figs 4 and 5, and Figs 9, 10, 11, and 12 establish the effectiveness of the proposed skull stripping algorithm over existing BET [41] in the proposed brain MR image segmentation method.

### Importance of Unsupervised Feature Selection

The proposed brain MR image segmentation method uses unsupervised feature selection algorithm based on the MRMS criterion to reduce the dimensionality of the data sets. Using the feature selection algorithm, the feature dimension can be reduced from *d* to *m* using (15). In order to establish the importance of unsupervised feature selection algorithm, extensive experimentation is carried out and the corresponding results are reported in Figs 9, 10, and 11. The performance of the proposed feature selection method is also compared with that of the feature selection algorithm proposed by Huang and Aviyente [50]. The eleventh, fourth, and fifth columns of heat maps of Figs 9, 10, and 11 compare the performance of the proposed method, ℳ_4_, and ℳ_5_, respectively.

From the results reported in fourth, fifth, and eleventh columns of heat maps of Figs 9, 10, and 11, it is seen that the proposed method achieves better results than ℳ_4_ in 25, 25, and 24 cases with respect to Jaccard index, sensitivity, and specificity, respectively, while the performance of the proposed method is better than that of ℳ_5_ in 20, 15, and 18 cases, respectively. Out of total 75 cases, the proposed method attains better results in 52 cases, while the methods ℳ_4_ and ℳ_5_ achieve it only in 1 and 22 cases, respectively. The second, sixth, and seventh columns of Figs 4 and 5 depict the comparative performance analysis of three methods qualitatively. All the results reported in Figs 4, 5, 9, 10, and 11 confirm that the unsupervised feature selection algorithm is effective in reducing the dimension of the feature space without losing segmentation quality. The better performance of the proposed unsupervised method over existing feature selection algorithm of Huang and Aviyente [50] is achieved due to the fact that the proposed method selects features based on their individual relevance as well as significance, whereas the method of Huang and Aviyente [50] considers only redundancy or similarity among them without considering their relevance values. In effect, the existing method selects a set of nonrelevant features, which degrades the quality of segmented images.

### Importance of Robust Rough-Fuzzy C-Means

Further, the performance of the proposed method is extensively compared with that of the methods ℳ_6_, ℳ_7_, and ℳ_8_. The only difference among these methods is the clustering algorithm used. While the proposed method uses robust rough-fuzzy *c*-means (rRFCM) [40] algorithm, other methods, namely, ℳ_6_, ℳ_7_, and ℳ_8_, use hard *c*-means (HCM) [54], fuzzy *c*-means (FCM) [35], and rough-fuzzy *c*-means (RFCM) [39], respectively.

The sixth, seventh, and eleventh columns of heat maps reported in Figs 9, 10, and 11 compare the performance of the proposed method with that of the methods ℳ_6_, ℳ_7_, and ℳ_8_ with respect to three quantitative indices on several brain MR images. The second, third, fourth, and fifth columns of Figs 6 and 7 compare the performance of different methods qualitatively. All the results reported in Figs 9, 10, and 11 establish the fact that the proposed method attains better performance in 18, 10, and 17 cases compared to other methods with respect to Jaccard index, sensitivity, and specificity, respectively, while the method ℳ_8_ achieves it in 7, 3, and 2 cases, respectively. On the other hand, the method ℳ_7_ provides better performance in 12 and 3 cases with respect to sensitivity and specificity, respectively, while the method ℳ_6_ attains highest specificity in only 3 cases. Out of total 75 cases, the proposed method achieves better performance in 45 cases, irrespective of the brain MR images and quantitative indices used. From the segmented images reported in second, third, fourth, and fifth columns of Figs 6 and 7, it can also be seen that there is a significant improvement in the segmentation results obtained using the proposed method as compared to other methods. In this regard, it should be noted that some of the existing clustering algorithms such as possibilistic *c*-means [37] fail to produce multiple segments of the input image as they generate coincident clusters even when they are initialized with final prototypes of the hard *c*-means.

The best performance of the proposed method is achieved due to the fact that the probabilistic membership function of the rRFCM handles efficiently overlapping partitions, while the possibilistic membership function of lower approximation of a cluster helps to discover arbitrary shaped cluster. Moreover, the concept of possibilistic lower approximation and fuzzy boundary of the rRFCM algorithm deals with uncertainty, vagueness, and incompleteness in class definition. In effect, good segmented regions are obtained using the proposed brain MR image segmentation algorithm.

### Comparative Performance Analysis

Finally, the performance of the proposed method is compared with that of both FSL [56] and SPM [57]. Results are reported in Figs 9, 10, and 11 with respect to Jaccard index, sensitivity, and specificity on both BrainWeb and IBSR data sets, while the qualitative performance analysis is reported in second, sixth, and seventh columns of Figs 6 and 7. The segmented outputs generated by the proposed method, FSL, and SPM, establish the fact that the proposed method generates more promising outputs than that obtained using the existing FSL and SPM. From the results reported in ninth, tenth, and eleventh columns of heat maps reported in Figs 9, 10, and 11, it can be seen that the proposed method provides better results than the FSL in 13, 6, and 11 cases, out of 13 cases each, for BrainWeb data set and in 10, 4, and 6 cases, out of 12 cases each, for IBSR data set with respect to Jaccard index, sensitivity, and specificity, respectively. In brief, the proposed method attains better performance than the FSL in 23, 10, and 17 cases, respectively, irrespective of the data sets used. On the other hand, the proposed method performs better than the SPM in 16 cases with respect to Jaccard index, while the performance of the proposed method detoriates compared to the SPM with respect to both sensitivity and specificity. The SPM achieves better results than the proposed method in 9, 24, and 19 cases, out of 25 cases each, with respect to Jaccard index, sensitivity, and specificity, respectively.

The better performance of the proposed method with respect to Jaccard index and lower sensitivity values obtained using the proposed method indicate that the proposed method attains lower ratio of false positive to true positive, which leads to lower false discovery rate (FDR), compared to both FSL and SPM in most of the cases, irrespective of the images used. The FDR is a multiple-hypothesis testing error measure indicating the expected proportion of false positives among the set of significant results. The FDR is particularly useful in the analysis of high-throughput data such as MRI. Out of total 25 cases, the proposed method attains lower FDR values in 17 and 23 cases than SPM and FSL, respectively. Moreover, the performance of the proposed method is compared with that of both FSL and SPM with respect to likelihood ratio positive (LR+), which is defined as the ratio of sensitivity and (1—specificity). Out of total 25 cases, the proposed method achieves higher LR+ values in 16 cases than the FSL, while SPM attains higher LR+ values in 19 cases than the proposed method.

The comparative performance analysis is also reported in terms of p-value computed through the sign test. The proposed method attains p-value of 9.72E-006, which is statistically significant considering 0.05 as the level of significance, for both Jaccard index and FDR with respect to the FSL. Also, it achieves lower p-value of 5.39E-02 for specificity and FDR, and that of 1.15E-01 for LR+ and Jaccard index, with respect to FSL and SPM, respectively. On the other hand, the SPM provides significant p-values of 2.98E-08 for sensitivity and 2.04E-03 for both specificity and LR+ with respect to the proposed method, while the FSL attains lower p-value of 1.15E-01 for sensitivity.

## Conclusion

The contribution of the paper lies in developing a methodology for brain MR image segmentation, which integrates judiciously a skull stripping method, dyadic wavelet analysis, unsupervised feature selection algorithm, and rough-fuzzy clustering algorithm. This formulation is geared towards maximizing the utility of rough-fuzzy clustering with respect to brain MR image segmentation tasks. Several quantitative measures are used to evaluate the performance of the proposed method. Finally, the effectiveness of the proposed method is demonstrated both qualitatively and quantitatively, along with a comparison with other related algorithms, on a set of synthetic as well as real life brain MR images. Although the methodology of integrating mask generation, wavelet analysis, unsupervised feature selection, and rough-fuzzy clustering has been efficiently demonstrated for brain MR images, the concept can be applied to other segmentation problems.

## References

[1] Suetens P (2002) Fundamentals of Medical Imaging, Cambridge University Press.

[2] Rangayyan RM (2004) Biomedical Image Analysis, CRC Press.

[3] Rosenfeld A, Kak AC (1982) Digital Picture Processing, Academic Press, Inc.

[4] Bezdek JC, Hall LO, Clarke LP (1993) Review of MR Image Segmentation Techniques Using Pattern Recognition. Medical Physics 20: 1033–1048. 10.1118/1.597000 8413011

[5] Haralick R, Shapiro L (1985) Survey: Image Segmentation Techniques. Computer Vision, Graphics and Image Processing 29: 100–132. 10.1016/S0734-189X(85)90153-7

[6] Maji P, Kundu MK, Chanda B (2008) Second Order Fuzzy Measure and Weighted Co-Occurrence Matrix for Segmentation of Brain MR Images. Fundamenta Informaticae 88: 161–176.

[7] Otsu N (1979) A Threshold Selection Method from Gray Level Histogram. IEEE Transactions on System, Man, and Cybernetics 9: 62–66. 10.1109/TSMC.1979.4310076

[8] Pal NR, Pal SK (1993) A Review on Image Segmentation Techniques. Pattern Recognition 26: 1277–1294. 10.1016/0031-3203(93)90135-J

[9] Sahoo PK, Soltani S, Wong AKC, Chen YC (1988) A Survey of Thresholding Techniques. Computer Vision, Graphics, and Image Processing 41: 233–260. 10.1016/0734-189X(88)90022-9

[10] Pal SK, King RA, Hashim AA (1983) Automatic Gray Level Thresholding Through Index of Fuzziness and Entropy. Pattern Recognition Letters 1: 141–146. 10.1016/0167-8655(83)90053-3

[11] Bovik AC, Clark M, Geisler WS (1990) Multichannel Texture Analysis Using Localized Spatial Filters. IEEE Transactions on Pattern Analysis and Machine Intelligence 12: 55–73. 10.1109/34.41384

[12] Chen CC, Chen DC (1996) Multiresolution Gabor Filters in Texture Analysis. Pattern Recognition Letters 17: 1069–1076. 10.1016/0167-8655(96)00065-7

[13] Jain AK, Farrokhnia F (1991) Unsupervised Texture Segmentation Using Gabor Filters. Pattern Recognition 24: 1167–1186. 10.1016/0031-3203(91)90143-S

[14] Turner MR (1986) Texture Discrimination by Gabor Function. Biological Cybernetics 55: 71–82. 3801538 10.1007/BF00341922

[15] Reed TR, Wechsler H (1990) Segmentation of Textured Images and Gestalt Organization Using Spatial/Spatial Frequency Representation. IEEE Transactions on Pattern Analysis and Machine Intelligence 12 10.1109/34.41379

[16] Mallat S (1989) A Theory for Multiresolution Signal Decomposition: The Wavelet Representation. IEEE Transactions on Pattern Analysis and Machine Intelligence 11: 674–693. 10.1109/34.192463

[17] Fukuda S, Hirosawa H (1999) A Wavelet-Based Texture Feature Set Applied to Classification of Multifrequency Polarimetric SAR Images. IEEE Transactions on Geoscience and Remote Sensing 37: 2282–2286. 10.1109/36.789624

[18] Lindsay RW, Percival DB, Rothrock DA (1996) The Discrete Wavelet Transform and the Scale Analysis of the Surface Properties of Sea Ice. IEEE Transactions on Geoscience and Remote Sensing 34: 771–787. 10.1109/36.499782

[19] Unser M, Eden M (1989) Multiresolution Feature Extraction and Selection for Texture Segmentation. IEEE Transactions on Pattern Analysis and Machine Intelligence 11: 717–728. 10.1109/34.192466

[20] Unser M (1995) Texture Classification and Segmentation Using Wavelet Frames. IEEE Transactionson Image Processing 4: 1549–1560. 10.1109/83.469936 18291987

[21] Acharyya M, Kundu MK (2002) Document Image Segmentation Using Wavelet Scale-Space Features. IEEE Transactions on Circuits and Systems for Video Technology 12: 1117–1127. 10.1109/TCSVT.2002.806812

[22] Bullitt E, Gerig G, Pizer SM, Aylward SR (2003) Measuring Tortuosity of the Intracerebral Vasculature from MRA Images. IEEE Transactions on Medical Imaging 22: 1163–1171. 10.1109/TMI.2003.816964 12956271 PMC2430603

[23] Clark MC, Hall LO, Goldgof DB, Velthuizen R, Murtagh FR, Silbiger M (1998) Automatic Tumor Segmentation Using Knowledge-Based Techniques. IEEE Transactions on Medical Imaging 117: 187–201. 10.1109/42.700731 9688151

[24] Kaus M, Warfield S, Nabavi A, Black PM, Jolesz FA, Kikinis R (2001) Automated Segmentation of MR Images of Brain Tumors. Radiology 218: 586–591. 10.1148/radiology.218.2.r01fe44586 11161183

[25] Prastawa M, Bullitt E, Ho S, Gerig G (2004) A Brain Tumor Segmentation Framework Based on Outlier Detection. Medical Image Analysis 8: 275–283. 10.1016/j.media.2004.06.007 15450222

[26] Just M, Higer HP, Schwarz M, Bohl J, Fries G, Pfannenstiel P, et al (1998) Tissue Characterization of Benign Tumors: Use of NMR-Tissue Parameters. Magnetic Resonance Imaging 6: 463–472. 10.1016/0730-725X(88)90482-1 3185139

[27] Gibbs P, Buckley DL, Blackband SJ, Horsman A (1996) Tumor Volume Determination from MR Images by Morphological Segmentation. Physics in Medical and Biology 41: 2437–2446. 10.1088/0031-9155/41/11/014 8938037

[28] Velthuizen RP, Clarke LP, Phuphanich S, Hall LO, Bensaid AM, Arrington JA, et al (1995) Unsupervised Measurement of Brain Tumor Volume on MR Images. Journal of Magnetic Resonance Imaging 5: 594–605. 10.1002/jmri.1880050520 8574047

[29] Bonnie NJ, Fukui MB, Meltzer CC, Huang QS, Day RS, Greer PJ, et al (1999) Brain Tumor Volume Measurement: Comparison of Manual and Semiautomated Methods. Radiology 212: 811–816. 10.1148/radiology.212.3.r99se22811 10478251

[30] Fletcher-Heath LM, Hall LO, Goldgof DB, Murtagh FR (2001) Automatic Segmentation of Non-Enhancing Brain Tumors in Magnetic Resonance Images. Artifical Intelligence in Medicine 21: 43–63. 10.1016/S0933-3657(00)00073-7 11154873

[31] Nie J, Xue Z, Liu T, Young GS, Setayesh K, Guo L, et al (2009) Automated Brain Tumor Segmentation Using Spatial Accuracy-Weighted Hidden Markov Random Field. Computerized Medical Imaging and Graphics 33: 431–441. 10.1016/j.compmedimag.2009.04.006 19446435 PMC2739047

[32] Xie K, Yang J, Zhang ZG, Zhu YM (2005) Semi-Automated Brain Tumor and Edema Segmentation Using MRI. European Journal of Radiology 56: 12–19. 10.1016/j.ejrad.2005.03.028 16168259

[33] Prastawa M, Bullitt E, Moon N, Leemput KV, Gerig G (2003) Automatic Brain Tumor Segmentation by Subject Specific Modification of Atlas Priors. Academic Radiology 10: 1341–1348. 10.1016/S1076-6332(03)00506-3 14697002 PMC2430604

[34] Noreen N, Hayat K, Madani SA (2011) MRI Segmentation through Wavelets and Fuzzy C-Means. World Applied Sciences Journal 13: 34–39.

[35] Bezdek JC (1981) Pattern Recognition with Fuzzy Objective Function Algorithms, Plenum, New York.

[36] Barra V, Boire JY (2000) Tissue Segmentation on MR Images of the Brain by Possibilistic Clustering on a 3D Wavelet Representation. Journal of Magnetic Resonance Imaging 11: 267–278. 10.1002/(SICI)1522-2586(200003)11:3<267::AID-JMRI5>3.0.CO;2-8 10739558

[37] Krishnapuram R, Keller JM (1993) A Possibilistic Approach to Clustering. IEEE Transactions on Fuzzy Systems 1: 98–110. 10.1109/91.227387

[38] Maji P, Pal SK (2012) Rough-Fuzzy Pattern Recognition: Applications in Bioinformatics and Medical Imaging. Wiley-IEEE Computer Society Press, New Jersey.

[39] Maji P, Pal SK (2007) Rough Set Based Generalized Fuzzy C-Means Algorithm and Quantitative Indices. IEEE Transactions on System, Man, and Cybernetics, Part B: Cybernetics 37: 1529–1540. 10.1109/TSMCB.2007.906578 18179071

[40] Maji P, Paul S (2013) Rough-Fuzzy Clustering for Grouping Functionally Similar Genes from Microarray Data. IEEE/ACM Transactions on Computational Biology and Bioinformatics 10: 286–299. 10.1109/TCBB.2012.103 22848138

[41] Smith SM (2002) Fast Robust Automated Brain Extraction. Human Brain Mapping 17: 143–155. 10.1002/hbm.10062 12391568 PMC6871816

[42] Park JG, Lee C (2009) Skull Stripping Based on Region Growing for Magnetic Resonance Brain Images. NeuroImage 47: 1394–1407. 10.1016/j.neuroimage.2009.04.047 19389477

[43] Adams R, Bischof L (1994) Seeded Region Growing. IEEE Transactions on Pattern Analysis and Machine Intelligence 16: 641–647. 10.1109/34.295913

[44] Shattuck D, Sandor-Leahy S, Schaper K, Rottenberg D, Leahy R (2001) Magnetic Resonance Image Tissue Classification Using a Partial Volume Model. NeuroImage 13: 856–876. 10.1006/nimg.2000.0730 11304082

[45] Atkins MS, Mackiewich BT (1998) Fully Automatic Segmentation of the Brain in MRI. IEEE Transactions on Medical Imaging 17: 98–107. 10.1109/42.668699 9617911

[46] Gao J, Xie M (2009) Skull-Stripping MR Brain Images using Anisotropic Diffusion Filtering and Morphological Processing. In: Proceedings of the IEEE International Symposium on Computer Network and Multimedia Technology. pp. 1–4.

[47] Coifman R, Wickerhauser M (1992) Entropy-Based Algorithms for Best Basis Selection. IEEE Transactions on Information Theory 38: 713–718. 10.1109/18.119732

[48] Saito N, Coifman RR, Geshwind FB, Warner F (2002) Discriminant Feature Extraction Using Empirical Probability Density Estimation and A Local Basis Library. Pattern Recognition 35: 2841–2852. 10.1016/S0031-3203(02)00019-5

[49] Chang T, Kuo CC (1993) Texture Analysis and Classification with Tree-Structured Wavelet Transform. IEEE Transactions on Image Processing 2: 429–441. 10.1109/83.242353 18296228

[50] Huang K, Aviyente S (2006) Information-Theoretic Wavelet Packet Subband Selection for Texture Classification. Signal Processing 86: 1410–1420. 10.1016/j.sigpro.2005.07.032

[51] Meyer FG, Chinrungrueng J (2003) Analysis of Event-Related fMRI Data Using Best Clustering Bases. IEEE Transactions on Medical Imaging 22: 933–939. 10.1109/TMI.2003.815869 12906247

[52] Huang K, Aviyente S (2008) Wavelet Feature Selection for Image Classification. IEEE Transactions on Image Processing 17: 1709–1720. 10.1109/TIP.2008.2001050 18713675

[53] Maji P, Paul S (2011) Rough Set Based Maximum Relevance-Maximum Significance Criterion and Gene Selection from Microarray Data. International Journal of Approximate Reasoning 52: 408–426. 10.1016/j.ijar.2010.09.006

[54] McQueen J (1967) Some Methods for Classification and Analysis of Multivariate Observations. In: Proceedings of the 5th Berkeley Symposium on Mathematics, Statistics and Probability. pp. 281–297.

[55] Pawlak Z (1991) Rough Sets: Theoretical Aspects of Resoning About Data, Kluwer, Dordrecht, The Netherlands.

[56] Smith SM, Jenkinson M, Woolrich MW, Beckmann CF, Behrens TE, Johansen-Berg H, et al (2004) Advances in Functional and Structural MR Image Analysis and Implementation as FSL. NeuroImage 23: 208–219. 10.1016/j.neuroimage.2004.07.051 15501092

[57] Ashburner J, Friston KJ (2005) Unified Segmentation. NeuroImage 26: 839–851. 10.1016/j.neuroimage.2005.02.018 15955494

