# Correction: Rough-Fuzzy Clustering and Unsupervised Feature Selection for Wavelet Based MR Image Segmentation

**DOI:** 10.1371/journal.pone.0132081

**Published:** 2015-06-29

**Authors:** The PLOS ONE Staff

There are a number of errors in the axes labels for Fig 8, “Heat maps for comparative performance analysis of different decomposition levels of wavelet analysis (from left to right: Jaccard index, sensitivity, and specificity).” The publisher apologizes for the errors. Please see the corrected Fig 8 here.

**Fig 8:**
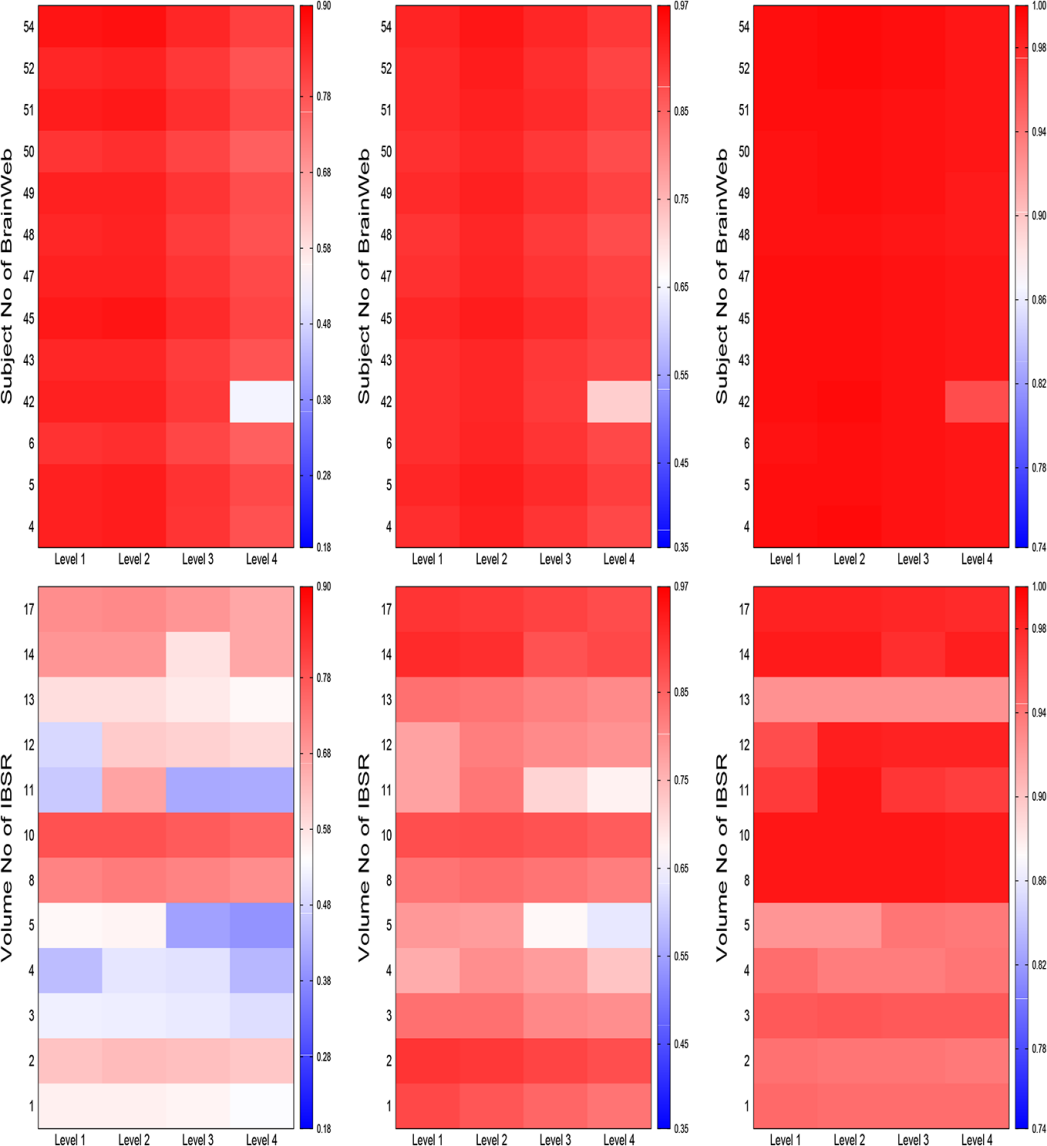
Heat maps for comparative performance analysis of different decomposition levels of wavelet analysis (from left to right: Jaccard index, sensitivity, and specificity).

There is an error in the axis label for Fig 9, “Heat maps obtained by different methods with respect to Jaccard index.” The publisher apologizes for the error. Please see the corrected Fig 9 here.

**Fig 9:**
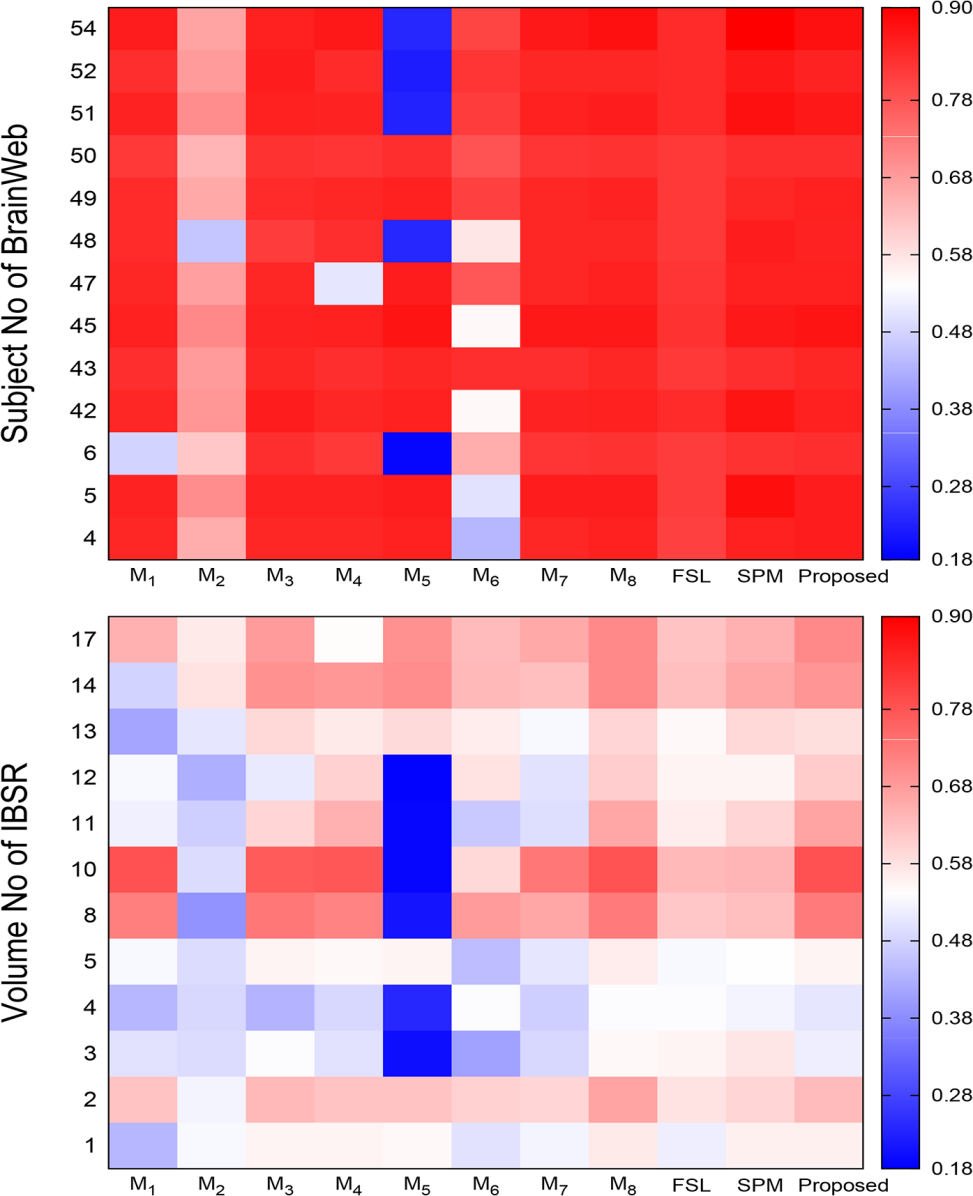
Heat maps obtained by different methods with respect to Jaccard index.

There is an error in the axis label for Fig 10, “Heat maps obtained by different methods with respect to sensitivity.” The publisher apologizes for the error. Please see the corrected Fig 10 here.

**Fig 10:**
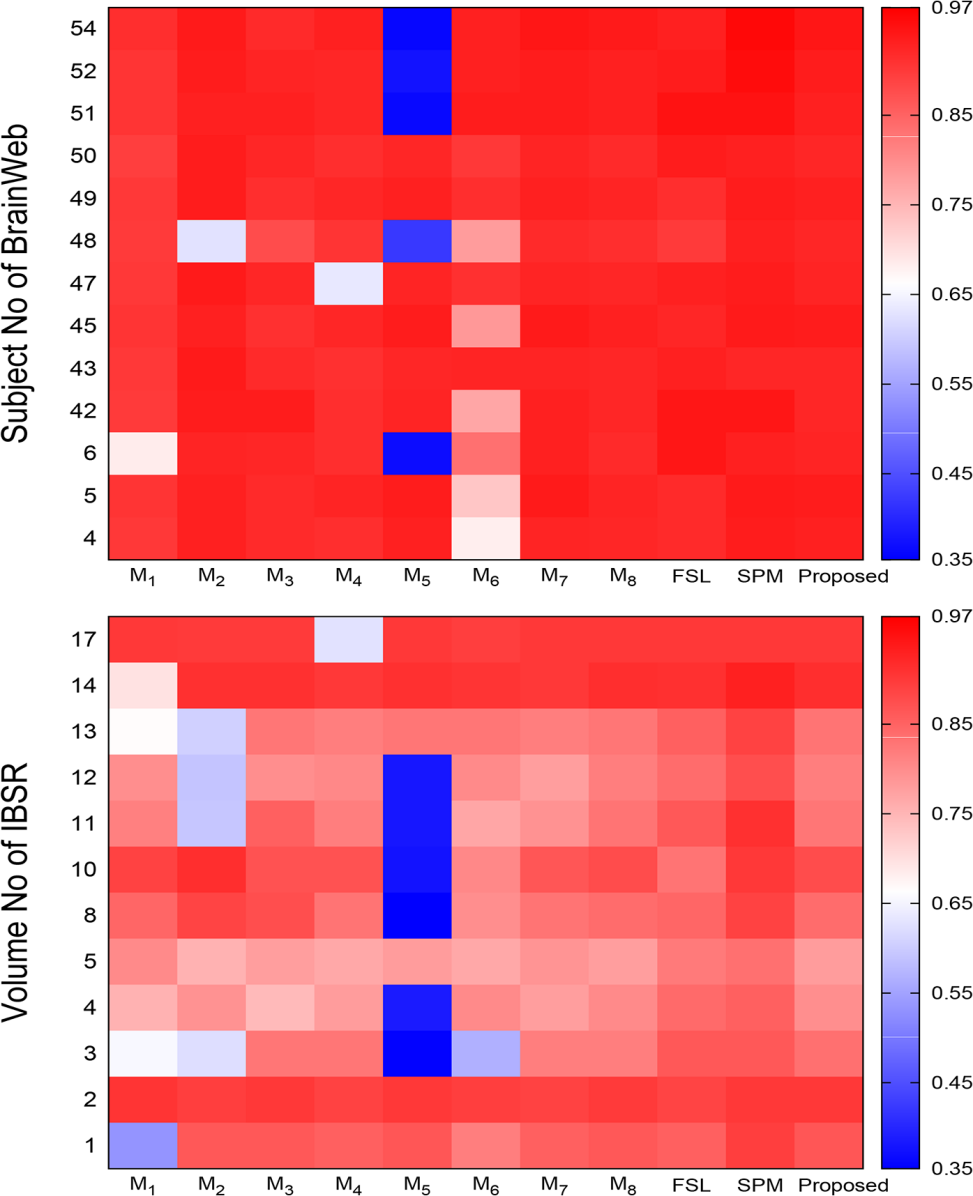
Heat maps obtained by different methods with respect to sensitivity.

There is an error in the axis label for Fig 11, “Heat maps obtained by different methods with respect to specificity.” The publisher apologizes for the error. Please see the corrected Fig 11 here.

**Fig 11:**
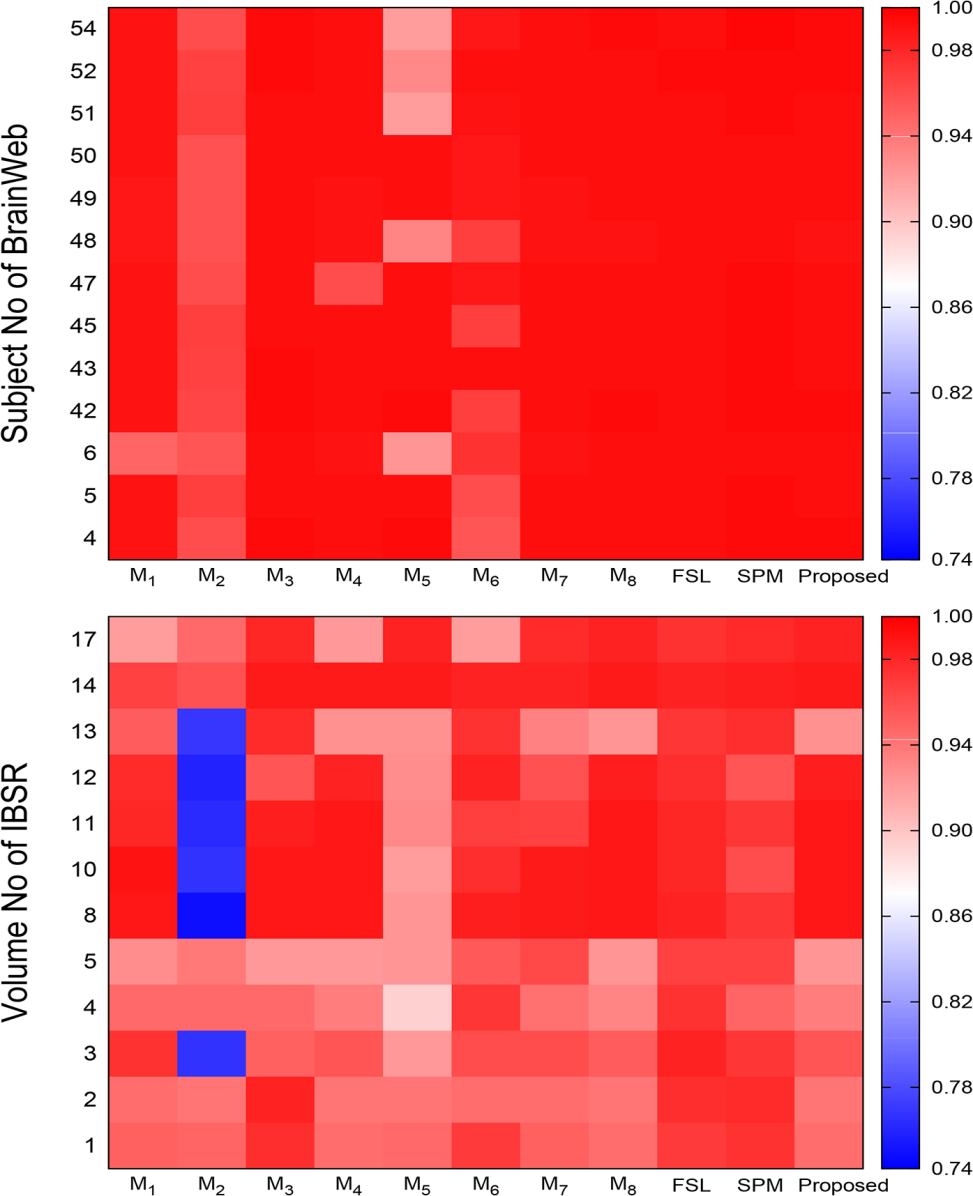
Heat maps obtained by different methods with respect to specificity.

There are a number of errors in the axes labels for Fig 12, “Heat maps for comparative performance analysis of the proposed method (skull stripping), the method ℳ2 (without skull stripping), and the method ℳ3 (masking using BET) for background separation (from left to right: Jaccard index, sensitivity, and specificity). The publisher apologizes for the errors. Please see the corrected Fig 12 here.

**Fig 12:**
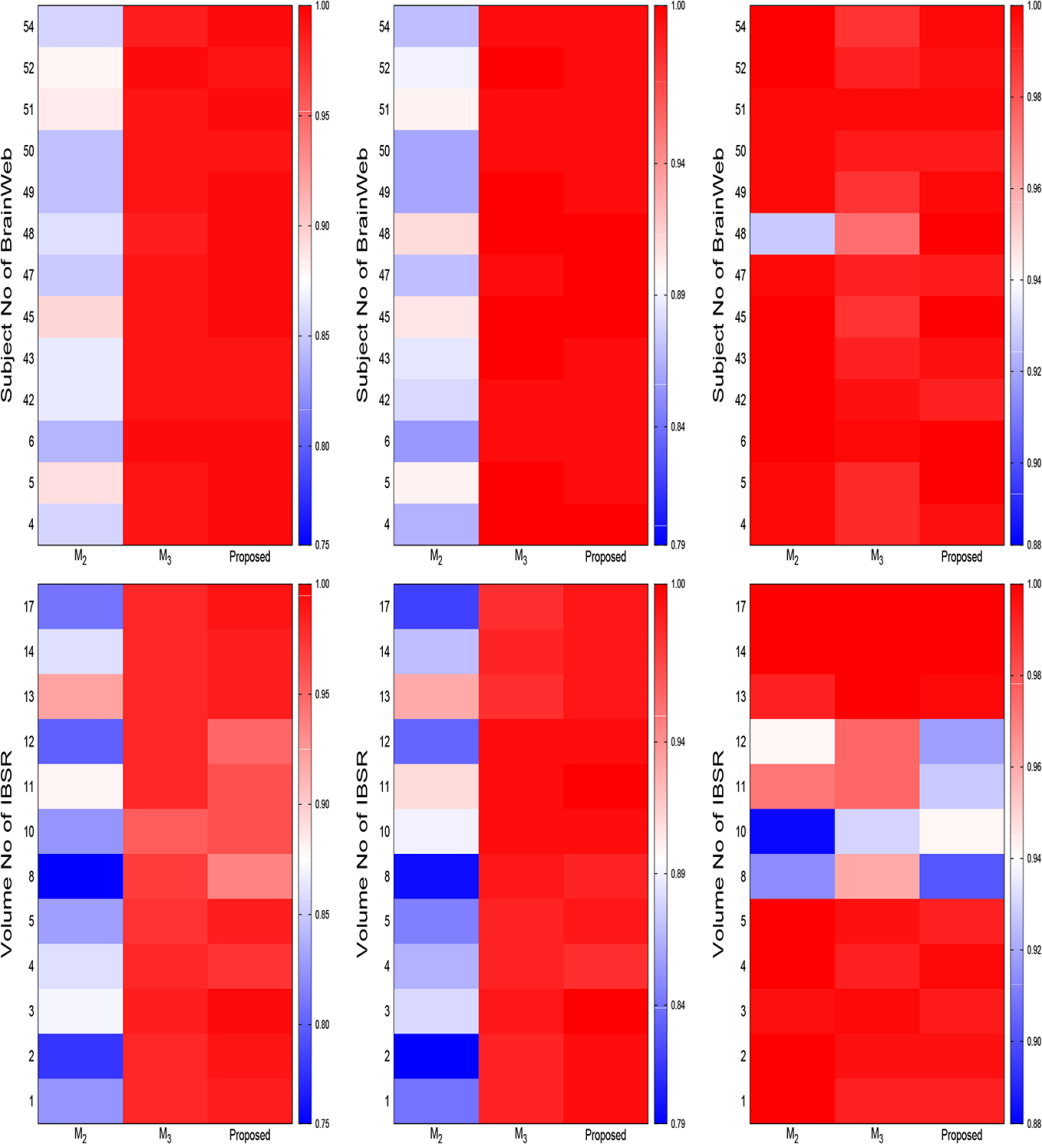
Heat maps for comparative performance analysis of the proposed method (skull stripping), the method ℳ2 (without skull stripping), and the method ℳ3 (masking using BET) for background separation (from left to right: Jaccard index, sensitivity, and specificity).
